# Repeated-dose toxicity and immunogenicity evaluation of a recombinant subunit COVID-19 vaccine (ZF2001) in rats

**DOI:** 10.3389/fcimb.2025.1548787

**Published:** 2025-04-22

**Authors:** Ruimin Sun, Lijuan Xia, Guangbiao She, Jinrong Li, Yiru Wang, Yunxiang Chen, Qian Yang, Siming Zhang, Fang Liu, Ying Chen, Liyan Zhang, Chengda Zhang, Wanqiang Lv, Enqi Huang, Lijiang Zhang

**Affiliations:** ^1^ Key Laboratory of Drug Safety Evaluation and Research of Zhejiang Province, Center of Safety Evaluation and Research, Hangzhou Medical College, Hangzhou, China; ^2^ Engineering Research Center of Novel Vaccine of Zhejiang Province, Hangzhou Medical College, Hangzhou, China; ^3^ Anhui Zhifei Longcom Biopharmaceutical Co., Ltd., HeFei, China; ^4^ Recombinant Vaccine Research and Development Joint Laboratory of Anhui Province, HeFei, China; ^5^ Faculty of Chinese Medicine, Macau University of Science and Technology, Macao, Macao SAR, China; ^6^ Qingshan Lake Science and Technology Innovation Center, Hangzhou Medical College, Hangzhou, China

**Keywords:** COVID-19, subunit protein vaccine, ZF2001, repeated-dose toxicity study, immune response

## Abstract

Coronavirus disease 19 (COVID-19), caused by severe acute respiratory syndrome coronavirus 2 (SARS-CoV-2), had given rise to a massive epidemic. Owing to the high morbidity and mortality of COVID-19 and the lack of effective therapies, safe and effective vaccination is the optimum choice for controlling this epidemic and preventing infection. The protein subunit vaccine ZF2001, which targets the receptor-binding domain (RBD) protein of SARS-CoV-2, has a significant protective effect against COVID-19. At the beginning of the COVID-19 epidemic, to promote the early approval of ZF2001 for clinical trials by the National Medical Products Administration of China (NMPA), a comprehensive evaluation of its toxicity *in vivo* was warranted. In the present study, a major part of the above series of studies, we evaluated the safety, immunogenicity and efficacy of the ZF2001 vaccine for the first time in adult Sprague Dawley (SD) rats. The male and female rats were administered three doses of the ZF2001 vaccine (25 μg or 50 μg NCP-RBD protein/dose, containing the aluminum-based adjuvant). The safety profile of ZF2001 was assessed by observing the general health status, local toxicity at the site of administration, immunotoxicity, immunogenicity, blood chemistry and hematology parameters in SD rats. In general, our results indicated that the ZF2001 vaccine did not induce significant systemic toxicity in rats, with a no-observed adverse effect level (NOAEL) of 50 μg NCP-RBD protein/rat. Moreover, the ZF2001 vaccine showed good immunogenicity by inducing the production of specific IgG antibodies in rats after three consecutive immunizations. In addition, histological examination revealed recoverable inflammatory changes in quadricep muscles and adjacent lymph nodes at the vaccine injection site. In summary, our systematic toxicology study proves the safety, tolerability and immunogenicity of the ZF2001 vaccine, which further supports the results of clinical trials of ZF2001.

## 1 Introduction

Coronavirus disease 19 (COVID-19), caused by severe acute respiratory syndrome coronavirus 2 (SARS-CoV-2), has become a public health emergency of international concern (Kevadiya et al., 2021). The clinical manifestations of COVID-19 infection can range from asymptomatic disease, to a mild influenza-like illness, and even to life-threatening complications culminating in death (Coronaviridae Study Group of the International Committee on Taxonomy of, V, 2020). To date, the COVID-19 pandemic reported by the World Health Organization (WHO) has resulted in more than 772 million confirmed cases and more than 6 million deaths worldwide, making it a serious public health threat worldwide. Moreover, the continuous emergence of new variants or strains of SARS-CoV-2 further contributes to the increased infectivity, immune escape, and pathogenicity of this virus (Attaway et al., 2021; Tao et al., 2021). Therefore, to the continued search for safe and effective prophylactic vaccines against COVID-19 is urgently needed.

The spike protein of SARS-CoV-2 mediates its binding to angiotensin-converting enzyme 2 (ACE2), which is the primary mechanism of SARS-CoV-2 infection and COVID-19 pathogenesis (Jackson et al., 2022). The receptor binding domain (RBD), known as the region specific for spike-ACE2 protein–protein interactions that contains multiple predominantly neutralizing epitopes, is a key antigen target for the development of COVID-19 vaccines (Barnes et al., 2020). According to the WHO, multiple candidate vaccines are currently being tested in phase 2 and phase 3 clinical trials or are in the development pipeline (Yadav et al., 2023). These vaccines can induce antibodies specific for the RBD, such as mRNAs, replication-incompetent viral vectors, inactivated vaccines and protein subunit vaccines, which effectively neutralize pseudotyped and live SARS-CoV-2 infection (Sadarangani et al., 2021). Among these vaccines, the protein subunit vaccine can induce a Th1 cell response and increase the titer of neutralizing antibodies; moreover this vaccine has the advantages of high yield, safety, easy storage and transportation, which makes it one of the most important therapeutic agents for preventing and preventing the spread of COVID-19 (Arunachalam et al., 2021; Sadarangani et al., 2021). Notably, ZF2001, a protein subunit vaccine targeting the RBD, has been approved for emergency therapeutic use in China, Uzbekistan, Indonesia, and Columbia. In phase 1 (NCT04445194, NCT04550351) and phase 2 (NCT04466085) clinical trials of ZF2001, vaccination with 25 or 50 μg NCP-RBD protein/dose and two- or three-dose schedules exhibited safety and immunogenicity (Yang et al., 2021). In a phase 3 (NCT04646590) clinical trial, the vaccine efficacy of ZF2001 was 75.7%, and that for preventing severe-to-critical COVID-19 was 87.6% (Dai et al., 2022). Moreover, in a large cohort of adults with a full vaccination schedule, the ZF2001 vaccine was shown to be safe and effective against symptomatic and severe-to-critical COVID-19 for at least 6 months (Dai et al., 2022; Jin et al., 2022; Liao et al., 2022). On the other hand, the ZF2001 vaccine was reported to have protective efficacy in preclinical studies of mice and nonhuman primates, which is consistent with the results of human clinical trials (An et al., 2022).

Shortly after the COVID-19 epidemic in 2020, to promote the early approval of ZF2001 for clinical trials as soon as possible, our laboratory initiated a full suite of nonclinical safety evaluation studies of the ZF2001 vaccine. Here, we present one of the earliest major toxicity studies in SD rats according to Good Laboratory Practices (GLPs) (OECD, 1997) to detect possible adverse effects, immunogenicity, immunotoxicity, and toxicity in target organs at the same dose administered to humans. Therefore, the data from this study were effectively used to verify the safety of ZF2001 and directly led to the approval of its entry into clinical trials.

## 2 Materials and methods

### 2.1 Study design

**Figure 1:**
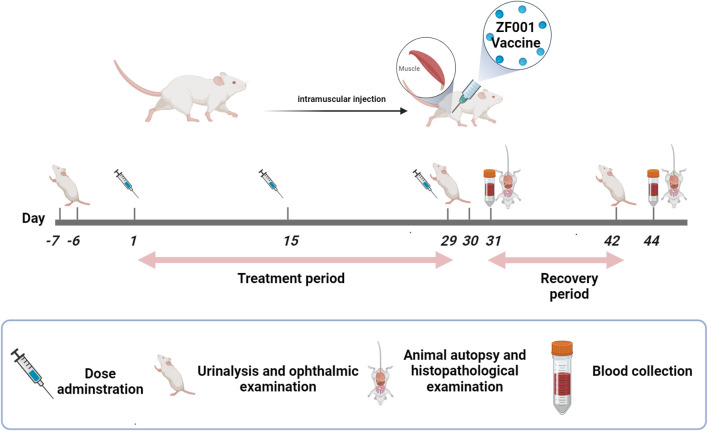
An overview of the safety evaluation of the ZF2001 vaccine.

The study design is presented in **Figure 1**. To determine the dose–response relationship and NOAEL, repeat toxicity of the ZF2001 vaccine was evaluated after 3 consecutive injections at 4 weeks into the treatment period and 2 weeks into the recovery period. In brief, 120 SD rats (60 males and 60 females) were randomly separated into four groups: a blank control group (0.9% sodium chloride solution), an adjuvant control group (aluminum-based adjuvant), a low-dose group (25 μg NCP-RBD protein/dose) and a high-dose group (50 μg NCP-RBD protein/dose). These dose settings were based on the related guidelines of the International Council for Harmonization of Technical Requirements for Pharmaceuticals for Human Use (ICH) and the National Medical Products Administration of China (NMPA) for nonclinical repeated-dose toxicity studies of vaccines (ICH, 2009; NMPA, 2010). The ZF2001 vaccine was developed by Anhui Zhifei Longcom Biopharmaceutical Co., Ltd. and the Institute of Microbiology, Chinese Academy of Sciences.

Specific-pathogen-free SD rats (males weighing 260–330 g and females weighing 179–234 g) were supplied by Shanghai SLAC Laboratory Animal Co., Ltd. (SCXK [Shanghai] 2017–0005; quality certificate No. 20170005023786). The study was conducted by the Center of Safety Evaluation and Research, Hangzhou Medical College (SYXK [Zhejiang] 2017-0010), which is an accredited organization of the Association for Assessment and Accreditation of Laboratory Animal Care International (AAALAC, #001489). SD rats were housed in single-sex groups (with a maximum of 5 rats per cage) and were provided with a complete rodent diet and locally sourced water that was softened and filtered *ad libitum*. The environmental conditions, including a relative humidity ranging from 47% to 67% and a temperature ranging from 21.5°C to 24.0°C, along with a 12 h light/dark cycle, were maintained throughout the study. All animal care and experimental procedures were conducted in compliance with the guidelines for the care and use of laboratory animals and the relevant regulations of the Institutional Animal Care and Use Committee (IACUC) and were approved by the IACUC (approval No. GLP-2020-030).

### 2.2 Clinical observation and routine measurement endpoints

During the administration period, the animals’ general health status, including their appearance, behavior, injection sites, skins and fur, was monitored every day. Body weights and food consumption were measured once a week. Hematological and serum biochemical analyses were conducted at both the end of the treatment period and the end of the recovery period for further analysis. The hematological indices were analyzed via an ADVIA-2120 Hematology Analyzer (Bayer, USA), and the serum biochemical indices were analyzed via a HITACHI7100 Automatic Biochemistry Analyzer (HITACHI, Japan), whose measured parameters are detailed in **Supplementary Table S2**. The coagulation indices were assayed via a Sysmex CA-1500 (Sysmex, Japan) for activated partial thromboplastin time (APTT), fibrinogen (Fbg), and prothrombin time (PT). Urinalysis and ophthalmic examinations were performed one week before vaccination, 24 h after the last dose, and 2 weeks after the last administration of the vaccine. The urine of each animal was collected in a metabolic cage. Urinalysis was carried out via a DIRUI N-600 (China), and the measured parameters are shown in **Supplementary Table S2**. Ophthalmic examination was carried out using Welch Allyn 12500 Binocular Indirect Inspection Eyewear (USA).

### 2.3 Immunogenicity and immunotoxicology evaluation

Serum samples from each rat used for the immunogenicity test were collected at both the end of the treatment period and the end of the recovery period. Immunogenicity was investigated via ELISA to determine the geometric mean titer (GMT) of the anti-NCP-RBD antibody (IgG). Briefly, 96-well microtiter ELISA plates were coated with 1 µg/mL (100 µL/well) whole inactivated SARS-CoV-2 antigen in 1x Coating Solution (PBS) and then incubated at 2~8°C overnight. After blocking with 3% w/v skim milk (Sangon Biotech, USA) in 1x PBST for 2 h at 37°C, the plates were washed three times with 1x PBST. Sera from immunized rats were twofold serially diluted in 3% w/v skim milk in PBST, added to the plates and incubated for 1 h at 37°C. Next, the plates were washed with 1x PBST and incubated with goat anti-rat IgG HRP-conjugated antibodies (Abcam, UK) at a dilution of 1:10000 for 1 h at 37°C. The binding of the secondary antibodies was visualized by adding TMB substrate solution. The enzymatic reaction was terminated by the addition of Elisa stopping solution. The absorbance at 450 nm (A_450_) was read on a microplate reader (Molecular Devices, Germany).

The levels of C3, C4 and IgG were detected by immunoturbidimetry with a HITACHI7100 automatic biochemical analyzer (Japan).

Peripheral blood lymphocytes were tested at the last dose of vaccination and 2 weeks after the last vaccination. In addition, the percentages of CD3^+^, CD4^+^ or CD8^+^ T lymphocytes and the ratio of CD4^+^/CD8^+^ T lymphocytes were measured in the peripheral blood.

### 2.4 Necropsy and histopathology

Ten SD rats per sex from each group were euthanized 2 days after receiving the 3rd dose, and the remaining animals were sacrificed 15 days after the last vaccination. Gross necropsy was performed immediately after each animal was euthanized. The weights and organ coefficients of the main organs, including the brain, heart, spleen, liver, kidney, adrenal gland, thymus, testicle (male rats), epididymis (male rats), ovary (female rats) and uterus (female rats), were tested for each rat. The main organs, including the heart, liver, spleen, lung, and kidneys, were preserved in 10% neutral buffered formalin. The eyes were preserved in Davidson’s fixative. The lungs were flushed with fixative at the time of necropsy. All preserved tissues were paraffin embedded, sectioned, stained with hematoxylin and eosin (H&E) and examined microscopically. Both smears (sternum for rats) and paraffin-embedded sternum sections were used to examine the bone marrow cellular morphology.

### 2.5 Statistical analysis

In this study, statistical analysis was performed via SPSS (version 18.0; New York, USA). Quantitative traits, including weight, growth gain rate, body temperature, hematology, biochemistry, electrocardiogram parameters, immunology indicators, organ weight and ratio, and safety pharmacology measurements, were summarized using the mean ± standard deviation (SD). Differences between multiple groups were analyzed using one-way ANOVA. The two groups were compared via the least significant difference (LSD) test if Levene’s test was not significant or the Games-Howell test if it was significant. Differences with *p* ≤ 0.05 were considered significant.

## 3 Results

### 3.1 Clinical observations

**Figure 2:**
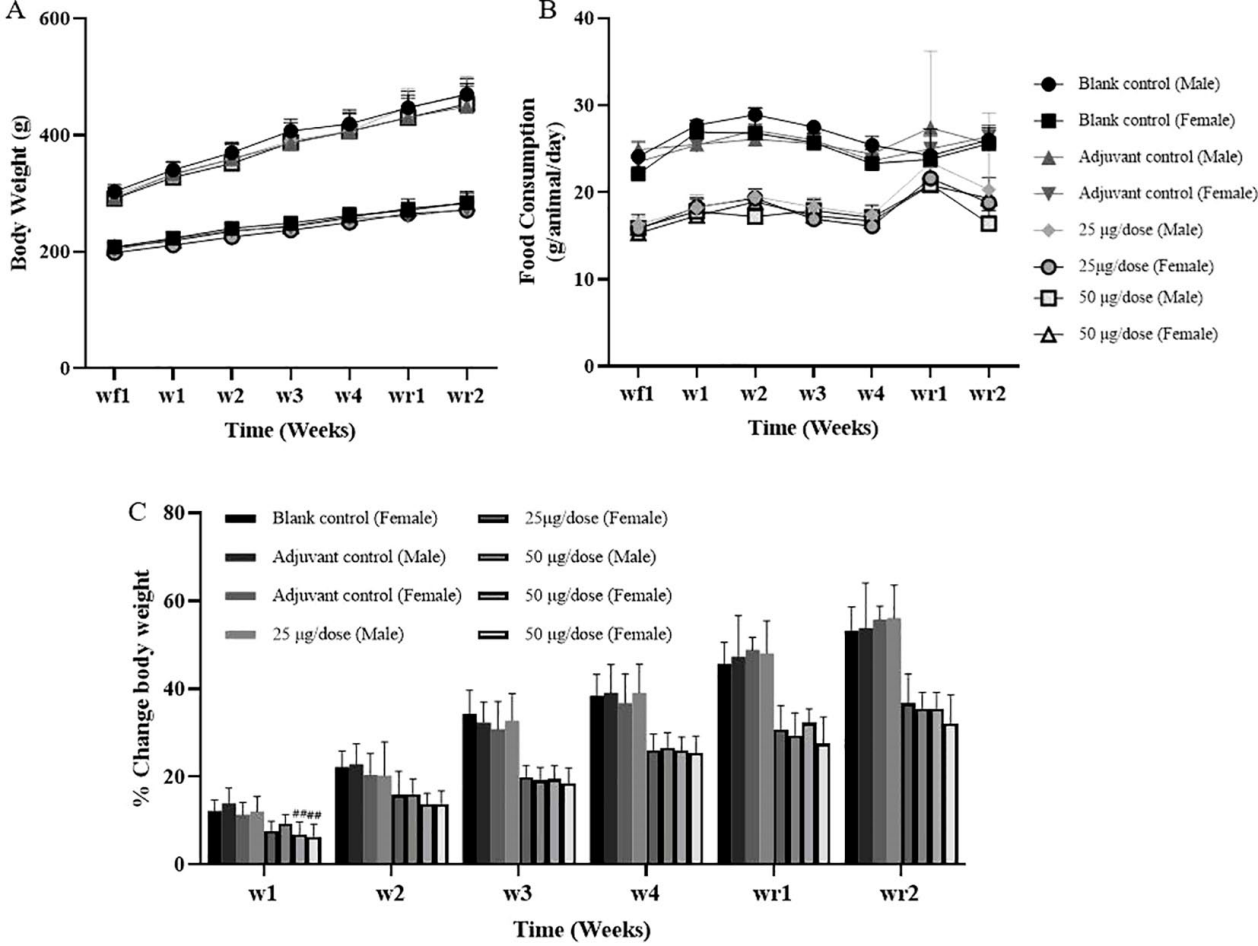
Body weights and food consumption of the rats. **(A)** Body weight, **(B)** Food consumption, **(C)** Body weight Change. Compared with the adjuvant control group, ^#^ *P*<0.05, ^##^ *P*<0.01, end of the dosing phase (n = 15/sex), end of the recovery period (n = 5/sex).

During the entire study, no deaths or obvious clinically abnormal symptoms were observed in any of the rats. All the rats in the adjuvant control and high-dose groups developed nodules at the administration sites, and these nodules persisted until the end of the recovery period, without complete resolution. The body weight of each group was not obviously affected, except that the body weight of female rats in the low-dose group at week 1 and week 2 was slightly lower than that of those in the blank control group; however, the body weight growth rates did not significantly differ, and there was no dose correlation; consequently, this was considered to indicate an occasional individual body weight fluctuation with no obvious toxicological significance (**Figures 2A–C**, **Supplementary Tables S3**, **S4**). Moreover, the food intake in all experimental groups remained largely comparable throughout the study. However, a marginal reduction in food intake was observed in female rats from the low-dose group during week 2, where values were slightly lower than those in the blank and adjuvant control groups. This reduction was minimal, limited to a single time point, and exhibited no discernible dose-relatedness, which were considered individual fluctuations of no obvious toxicological significance (**Supplementary Table S5**).

### 3.2 Hematological and serum biochemical analysis

Compared with the blank control group, the adjuvant control group, low-dose group, and high-dose group presented decreased changes in coagulation-related indices (PT, APTT, Fbg) and increased mean platelet volume (MPV) (**Table 1**). Considering the similar changes in the adjuvant control group, the decrease in coagulation-related indices was small (the average decrease was less than 15%) and within the normal reference value of our laboratory; furthermore, the decrease was considered to be related to the intramuscular injection of aluminum-containing adjuvants but not to the role of antigenic proteins in the vaccine. The number of EOSs in the low- and high-dose groups was greater than that in the adjuvant control group in the two-phase examination, which may have been related to the inflammatory immune response caused by vaccination. The results of the hematological analysis are detailed in **Table 1**.

**Table 1:** The hematology analysis in SD rats treated with ZF2001 vaccines.

Parameter	End of the dosing phase (n = 20)				End of the recovery period (n = 10)			
	Blank control	Adjuvant control	Low-dose	High-dose	Blank control	Adjuvant control	Low-dose	High-dose
WBC (10e^3^/μL)	6.37 ± 3.01	5.15 ± 1.85	4.99 ± 1.35	5.54 ± 1.30	6.50 ± 2.23	5.49 ± 1.86	5.52 ± 2.13	5.54 ± 1.42
%NEUT (%)	14.8 ± 6.7	14.2 ± 3.8	17.3 ± 9.6	14.7 ± 5.6	12.3 ± 6.0	11.0 ± 2.5	11.9 ± 5.5	13.0 ± 5.1
%LYMPH (%)	80.0 ± 7.4	80.2 ± 4.1	76.6 ± 10.3	78.7 ± 6.2	81.6 ± 6.2	84.0 ± 2.4	81.8 ± 5.4	80.1 ± 5.6
%MONO (%)	2.3 ± 0.9	2.7 ± 0.6	2.6 ± 0.9	2.9 ± 1.0	2.9 ± 0.7	2.2 ± 0.6	2.6 ± 0.9	2.9 ± 1.0
%EOS (%)	1.8 ± 0.5	1.7 ± 0.5	2.3 ± 0.8^△^	2.3 ± 0.8*^△^	2.0 ± 0.9	1.6 ± 0.3	2.6 ± 0.8^△^	2.8 ± 1.0 *^△△^
%BASO (%)	0.2 ± 0.1	0.2 ± 0.1	0.2 ± 0.1	0.2 ± 0.0	0.2 ± 0.1	0.2 ± 0.1	0.2 ± 0.1	0.1 ± 0.1
%LUC (%)	1.0 ± 0.3	1.0 ± 0.3	1.0 ± 0.4	1.3 ± 0.5	1.0 ± 0.4	1.0 ± 0.3	0.9 ± 0.2	1.0 ± 0.3
#NEUT (10e^3^/μL)	0.99 ± 0.71	0.74 ± 0.36	0.86 ± 0.57	0.85 ± 0.48	0.82 ± 0.46	0.63 ± 0.30	0.74 ± 0.61	0.74 ± 0.41
#LYMPH (10e^3^/μL)	5.04 ± 2.31	4.14 ± 1.49	3.83 ± 1.16	4.33 ± 0.93	5.30 ± 1.89	4.59 ± 1.52	4.44 ± 1.49	4.41 ± 1.04
#MONO (10e^3^/μL)	0.15 ± 0.10	0.13 ± 0.05	0.13 ± 0.05	0.16 ± 0.07	0.18 ± 0.06	0.12 ± 0.04	0.15 ± 0.08	0.16 ± 0.07
#EOS (10e^3^/μL)	0.11 ± 0.06	0.08 ± 0.03	0.11 ± 0.04	0.12 ± 0.04^△△^	0.12 ± 0.04	0.09 ± 0.03	0.14 ± 0.05	0.16 ± 0.09^△△^
#BASO (10e^3^/μL)	0.01 ± 0.01	0.01 ± 0.01	0.01 ± 0.01	0.01 ± 0.01	0.01 ± 0.01	0.01 ± 0.01	0.01 ± 0.01	0.01 ± 0.00
#LUC (10e^3^/μL)	0.07 ±0.05	0.05 ± 0.02	0.05 ± 0.03	0.07 ± 0.03	0.06 ± 0.02	0.05 ± 0.02	0.05 ± 0.02	0.05 ± 0.02
RBC (10e^6^/μL)	7.89 ±0.84	7.36 ± 0.47	7.82 ± 0.52^△^	7.63 ± 0.59	7.59 ± 0.35	7.49 ± 0.56	7.69 ± 0.36	7.66 ± 0.43
HGB (g/L)	147 ±14	140 ± 9	142 ± 7	140 ± 8	141 ± 4	136 ± 6	142 ± 5	139 ± 7
HCT (%)	43.7 ±4.3	41.4 ± 2.6	42.8 ± 2.5	41.7 ± 2.9	42.6 ± 1.9	41.3 ± 2.7	43.0 ± 2.0	41.9 ± 2.4
MCV (fL)	55.5 ±1.4	56.2 ± 1.0	54.7 ± 1.7^△△^	54.8 ± 1.6^△△^	56.2 ± 1.1	55.2 ± 1.4	55.9 ± 1.5	54.6 ± 1.1
MCH (pg)	18.6 ±0.6	19.0 ± 0.6	18.3 ± 0.8^△△^	18.4 ± 0.7^△△^	18.7 ± 0.6	18.2 ± 0.8	18.5 ± 0.8	18.2 ± 0.5
MCHC (g/L)	335 ±6	338 ± 8	333 ± 7	336 ± 7	332 ± 8	329 ± 10	330 ± 8	333 ± 6
RDW (%)	11.0 ±0.4	11.4 ± 0.8	11.1 ± 0.5	11.0 ± 0.4	11.7 ± 0.7	11.7 ± 0.7	11.5 ± 0.8	11.8 ± 0.7
HDW (g/L)	23.7 ±2.2	24.7 ± 2.3	24.1 ± 2.1	24.4 ± 2.4	24.1 ± 3.1	23.5 ± 1.9	22.7 ± 1.7	24.0 ± 1.3
MPV (fL)	8.0 ±0.6	8.5 ± 0.6**	8.7 ± 0.6**	9.0 ± 0.7**	9.0 ± 0.4	9.1 ± 0.3	8.9 ± 0.5	9.0 ± 0.5
PLT (10e^3^/μL)	1020 ±87	985 ± 103	1021 ± 102	1023 ± 90	1016 ± 115	1041 ± 61	1005 ± 71	1044 ± 94
%RETIC (%)	2.61 ±0.38	2.65 ± 0.42	2.54 ± 0.52	2.64 ± 0.59	2.75 ± 0.48	2.29 ± 0.42	2.51 ± 0.45	2.44 ± 0.38
PT (s)	204.3±24.4	194.4 ± 29.2	197.5 ± 37.0	199.8 ± 37.7	208.8 ± 36.5	171.3 ± 32.8	194.0 ± 40.3	187.3 ± 33.9
Fbg (g/L)	10.4 ±1.0	9.6 ± 0.7**	9.5 ± 0.6**	9.5 ± 1.0**	10.1 ± 0.7	8.9 ± 1.0**	10.1 ± 0.9^△△^	8.8 ± 0.5**
APTT (s)	2.171±0.217	2.201 ± 0.231	2.077± 0.328	2.292 ± 0.233	2.053 ± 0.147	1.829 ± 0.089**	2.075 ± 0.150^△△^	1.762 ± 0.108**

Compared with the blank control group, ^*^ *P*<0.05, ^**^ *P*<0.01. Compared with the adjuvant control group, ^△^ *P*<0.05, ^△△^ *P*<0.01.

At the end of the dosing phase, the globulin (GLO) level in the high-dose group was elevated, and the albumin–globulin ratio (A/G) was decreased, as shown in **Table 2**. These changes were consistent with the trend of elevated immunoglobulin G (IgG) and complement C3 (C3) levels, which were dose-related and may have been related to the immune stimulation induced by vaccination and were considered an extension of the pharmacological effects of the vaccine. Examinations at the end of the recovery period revealed that the above-described abnormalities were reversed. After 3 doses of vaccination, sodium (Na^+^) and chloride (Cl^-^) ions were elevated in the adjuvant control group and the low- and high-dose groups (**Table 2**), and since similar changes were observed in the adjuvant control group and the magnitude of the changes was small, the differences were considered possibly due to adjuvant factors rather than vaccine-related toxicity. There were also a few individual biochemical indicators with small differential fluctuations and no apparent dose-relatedness, which were considered individual fluctuations of no obvious toxicological significance.

**Table 2:** The blood biochemistry analysis in SD rats treated with ZF001 vaccines.

Parameter	End of the dosing phase (n = 20)				End of the recovery period (n = 10)			
	Blank control	Adjuvant control	Low-dose	High-dose	Blank control	Adjuvant control	Low-dose	High-dose
ALT(IU/L)	27.22 ± 5.55	25.36 ± 4.07	26.33 ± 6.38	25.32 ± 5.28	24.92 ± 5.49	24.27 ± 5.35	24.36 ± 5.89	25.17 ± 5.30
AST(IU/L)	155.95 ± 50.34	147.47 ± 49.45	163.14 ± 54.23	141.24 ± 29.82	159.92 ± 21.33	135.38 ± 30.56	145.77 ± 17.83	143.58 ± 34.72
T.BIL^a^(umol/L)	0.527 ± 0.304	0.400 ± 0.237	0.475 ± 0.268	0.472 ± 0.275	0.544 ± 0.254	0.565 ± 0.146	0.512 ± 0.311	0.578 ± 0.147
ALP(IU/L)	88.02 ± 39.68	81.43 ± 23.98	89.35 ± 30.43	89.69 ± 32.72	83.46 ± 18.94	79.13 ± 33.37	78.84 ± 34.87	77.40 ± 31.63
CK(IU/L)	617.7 ± 210.8	583.7 ± 281.0	541.8 ± 225.3	426.0 ± 139.0 **	367.6 ± 54.4	287.2 ± 62.4	319.6 ± 58.6	311.7 ± 85.2
T.P(g/L)	59.67 ± 3.58	56.36 ± 3.61**	58.33 ± 2.71	60.58 ± 3.39^△△^	57.22 ± 3.20	59.07 ± 3.53	56.79 ± 3.33	59.07 ± 2.53
ALB(g/L)	38.68 ± 2.86	37.06 ± 2.75	37.52 ± 1.69	38.16 ± 2.29	37.99 ± 1.52	39.55 ± 2.71	38.74 ± 2.72	39.05 ± 1.69
GLO(g/L)	20.99 ± 2.18	19.30 ± 1.61**	20.82 ± 1.58	22.42 ± 2.13*^△△^	19.23 ± 2.94	19.52 ± 1.19	18.05 ± 0.96	20.03 ± 1.36
A/G	1.86 ± 0.21	1.93 ± 0.17	1.81 ± 0.12^△^	1.71 ± 0.17**^△△^	2.02 ± 0.28	2.03 ± 0.12	2.15 ± 0.14	1.96 ± 0.13
GLU(mmol/L)	5.563 ± 1.123	5.724 ± 1.212	4.909 ± 1.220	5.198 ± 0.764	3.759 ± 0.708	4.140 ± 0.405	3.915 ± 0.634	3.849 ± 0.760
BUN(mmol/L)	6.413 ± 0.692	6.904 ± 0.809	6.327 ± 1.041	6.843 ± 0.653	7.026 ± 0.979	7.986 ± 1.841	7.426 ± 1.989	8.216 ± 2.077
Crea(umol/L)	45.54 ± 4.77	43.13 ± 4.24	44.38 ± 4.07	43.56 ± 2.66	52.52 ± 4.45	55.82 ± 8.22	52.11 ± 6.19	53.69 ± 5.31
T.CHO(mmol/L)	1.640 ± 0.193	1.648 ± 0.242	1.719 ± 0.150	1.751 ± 0.229	1.540 ± 0.209	1.551 ± 0.215	1.661 ± 0.224	1.626 ± 0.202
TG(mmol/L)	0.610 ± 0.531	0.503 ± 0.213	0.538 ± 0.373	0.522 ± 0.318	0.363 ± 0.227	0.502 ± 0.255	0.561 ± 0.157*	0.323 ± 0.146
K^+^(mmol/L)	4.36 ± 0.45	4.25 ± 0.40	4.50 ± 0.45	4.41 ± 0.40	4.70 ± 0.23	4.57 ± 0.37	4.71 ± 0.28	4.70 ± 0.25
Na^+^(mmol/L)	147 ± 2	148 ± 1**	149 ± 1**^△^	151 ± 1**^△△^	148 ± 1	148 ± 1	148 ± 1	150 ± 1**^△△^
Cl^-^(mmol/L)	105.8 ± 2.0	107.4 ± 0.9**	106.0 ± 1.8^△△^	107.1 ± 1.5*	104.2 ± 1.1	105.0 ± 1.5	104.8 ± 1.2	105.5 ± 1.6
Ca(mmol/L)	2.30 ± 0.04	2.27 ± 0.08	2.28 ± 0.06	2.39 ± 0.13**^△△^	2.26 ± 0.04	2.29 ± 0.05	2.24 ± 0.03	2.28 ± 0.04

Compared with the blank control group, *P<0.05, **P<0.01. Compared with the adjuvant control group, ^△^ *P*<0.05, ^△△^ *P*<0.01. ^a^The T.BIL test results of some of the samples were negative (1 in the control group, 5 in the adjuvant group, 1 in the low-dose group and 1 in the high-dose group). The statistical analysis was conducted after the negative data were excluded. The samples were counted as follows: n=19 in the blank control group, n=15 in the adjuvant control group, n=19 in the low-dose group and n=19 in the high-dose group.

### 3.3 Urinalysis and ophthalmic examination

No effects related to the test treatment were observed in the urinary parameters of the rats in the low- and high-dose groups or the adjuvant control group (**Supplementary Tables S6**-**S11**).

Throughout the study, the eyes of the rats in all groups were in good condition, except for a vitreous hemorrhage in the right eye of one rat in the high-dose group at the time of the discontinuation examination; this hemorrhage resolved by the time of the recovery ophthalmologic examination. Given that the above abnormalities were recoverable and that the histopathological examination revealed no obvious pathological abnormalities in this eye, we considered this complication to be an episodic, dose-independent individual spontaneous abnormality (data not presented in the text).

### 3.4 Immunotoxicological evaluation

**Figure 3:**
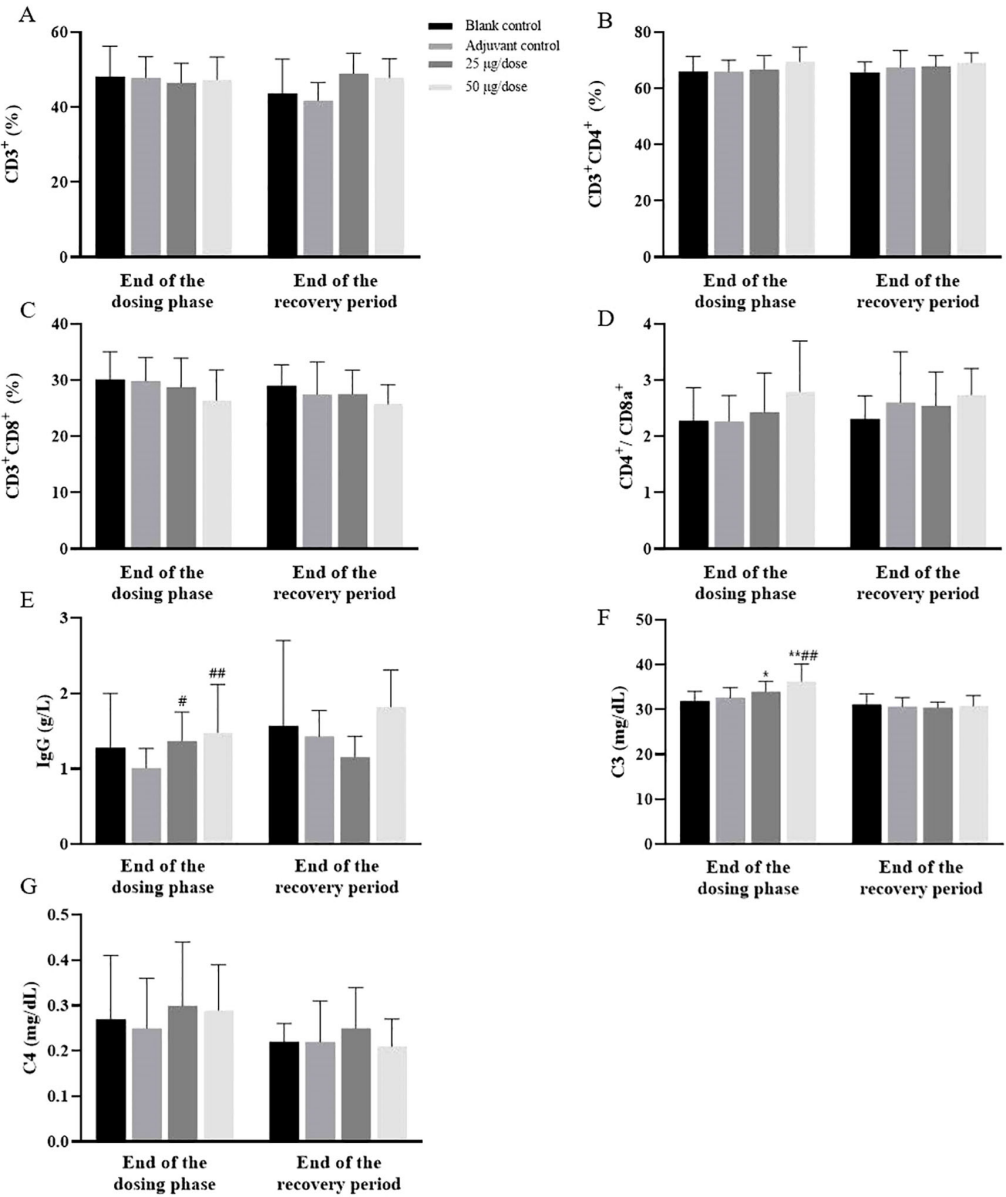
Changes in immunotoxicological indices in rats after 3 doses of vaccination. **(A–G)** The levels of CD3^+^, CD3^+^CD4^+^, CD3^+^CD8^+^, CD4^+^CD8a^+^, IgG, C3 and C4. Compared with the blank control group, ^#^ *P*<0.05, ^##^ *P*<0.01. Compared with the adjuvant control group, *P<0.05, **P<0.01, end of the dosing phase (n = 20), end of the recovery period (n = 10).

Compared with those of the blank control group, the immunophenotypes of the peripheral blood lymphocytes (CD3^+^, CD4^+^, and CD8^+^) of the rats in the low- and high-dose groups and the adjuvant control group were not significantly affected (**Figures 3A–D**). The levels of IgG and C3 in the low- and high-dose groups were greater than those in the blank control group at the end of the dosing phase, and both dosing groups recovered after 2 weeks of withdrawal (**Figures 3E, F**). No difference in C4 levels was observed between the groups (**Figure 3G**). The trend of increase in IgG and C3 was consistent with the trend of increase in GLO, which may have been related to the increase in the immune response caused by vaccination.

### 3.5 Immunogenicity

**Figure 4:**
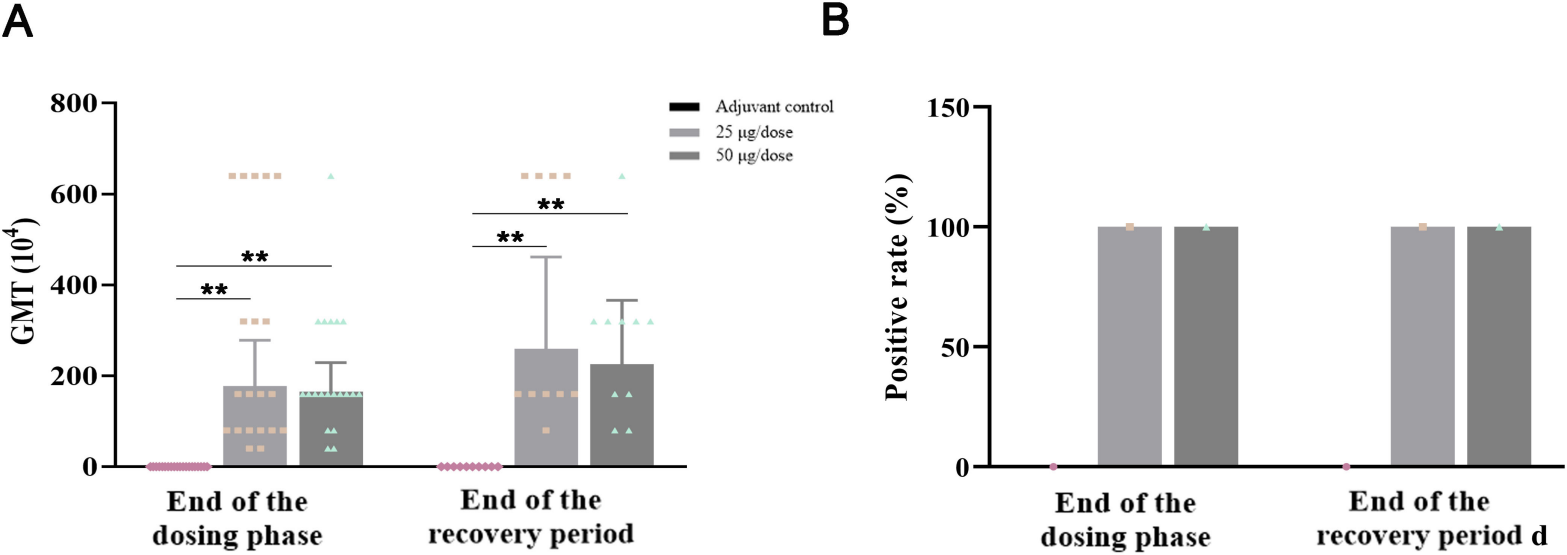
Immunogenicity results of ZF2001 after 3 doses of vaccination. **(A)** GMT of anti-NCP-RBD IgG antibodies. **(B)** Anti-NCP-RBD antibody positivity rates. Compared with the adjuvant control group, **P<0.01, end of the dosing phase (n = 20), end of the recovery period (n = 10).

In accordance with the immunization schedule, serum was collected from the animals, and antigen-specific IgG-binding antibodies against NCP-RBD were detected. The geometric mean titers (GMTs) of anti-NCP-RBD IgG antibodies in the low-dose and high-dose groups were 1.78×10^6^ and 1.66×10^6^, respectively, at the end of the dosing phase, whereas these values were 2.60×10^6^ and 2.26×10^6^, respectively, at the end of the recovery period (**Figure 4A**). At the end of the dosing phase and recovery period, the anti-NCP-RBD antibody positivity rate was 100% in both the low-dose and high-dose groups, whereas this rate was 0% in the adjuvant control group (**Figure 4B**). In conclusion, the above results indicate that ZF2001 has good immunogenicity and can induce specific anti-NCP-RBD IgG antibodies in rats after three consecutive injections.

### 3.6 Organ weights and organ coefficients

**Figure 5:**
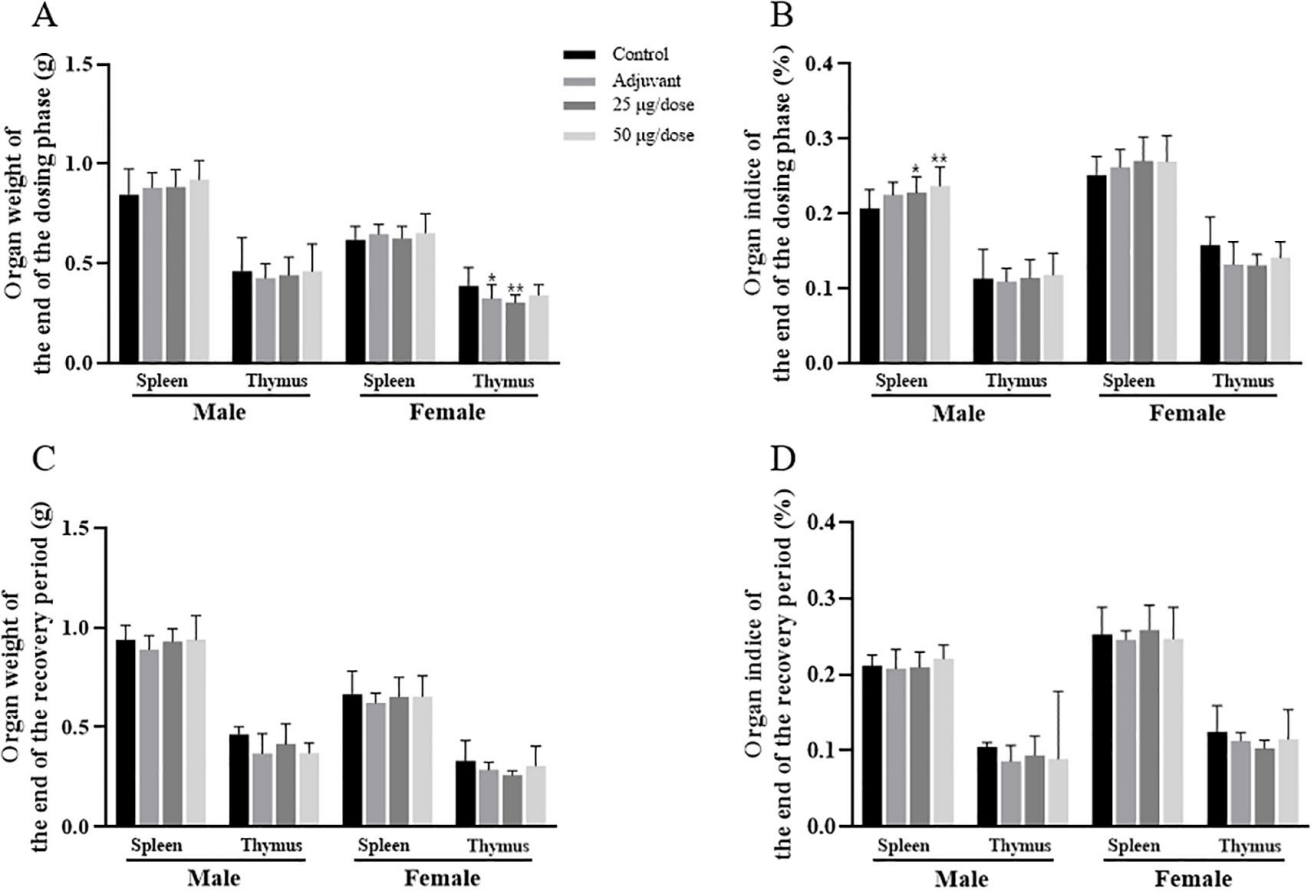
Organ weights and organ coefficients of the spleen and thymus in rats after 3 doses of vaccination. **(A–D)** Organ weights and organ coefficients. Compared with the blank control group, *P<0.05, **P<0.01, end of the dosing phase (n = 15/sex), end of the recovery period (n = 5/sex).

The organ weights and organ coefficients for each period are presented in **Figure 5** and **Supplementary Tables S12**-**S15**. At the end of the dosing phase, the organ coefficient of the spleen was greater in both the low-dose and high-dose groups of male rats than in the blank control group, which may have been related to the immune response after vaccination. Compared with that in the blank control group, the thymic weights in the female adjuvant control and low-dose groups were lower, which may have been related to animal stress after vaccination or individual fluctuations of no toxicological significance. At the end of the recovery period, no significant abnormalities in organ weights or coefficients were observed in either male or female rats in the low-dose group, the high-dose group and the adjuvant control group.

### 3.7 Necropsy and histopathology

**Figure 6:**
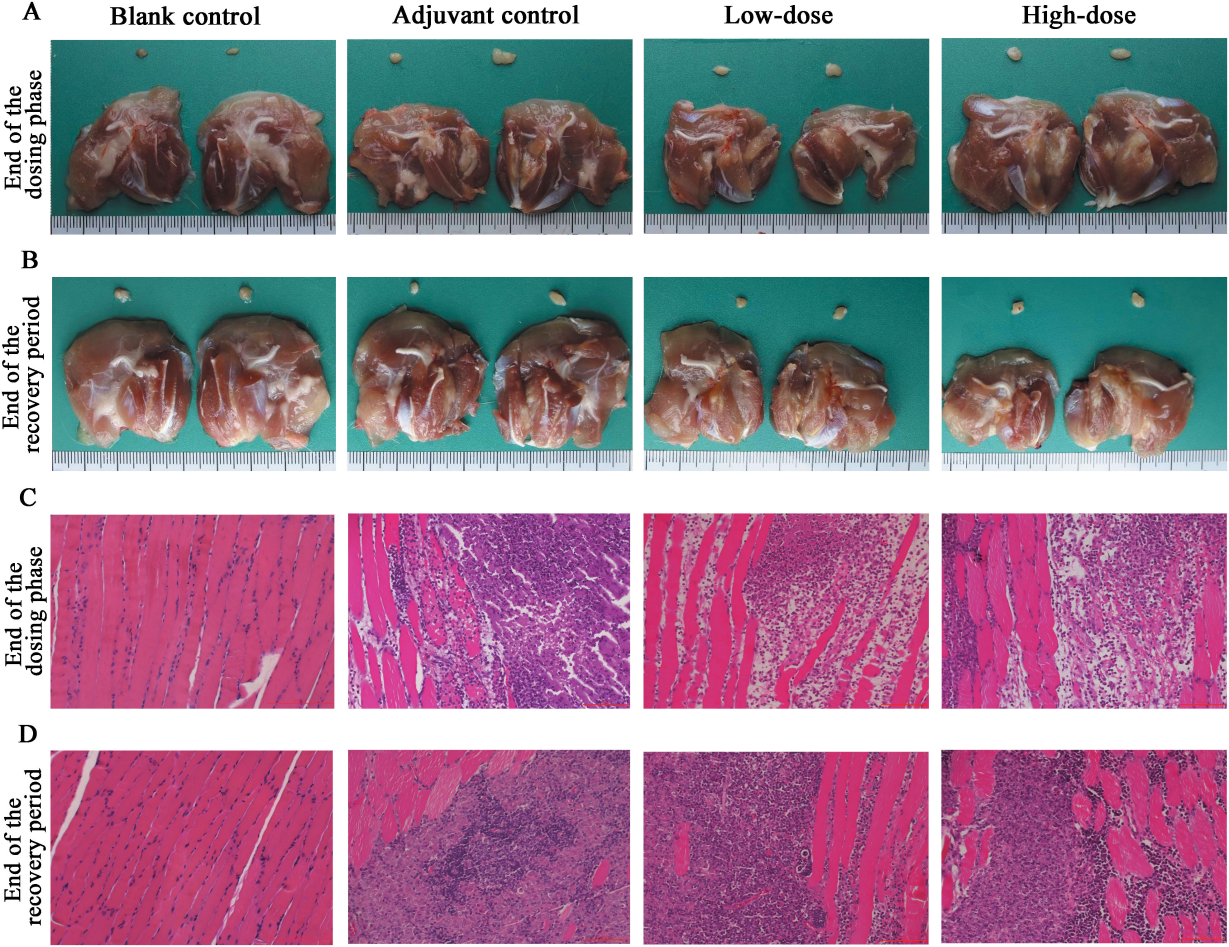
Histopathological results at the injection site of ZF2001 or the adjuvant control in rats. **(A, B)** are dissection photographs, and **(C, D)** are microscopic observation pictures (200×, scale bar=100 μm), end of the dosing phase (n = 20), end of the recovery period (n = 10).

**Figure 7:**
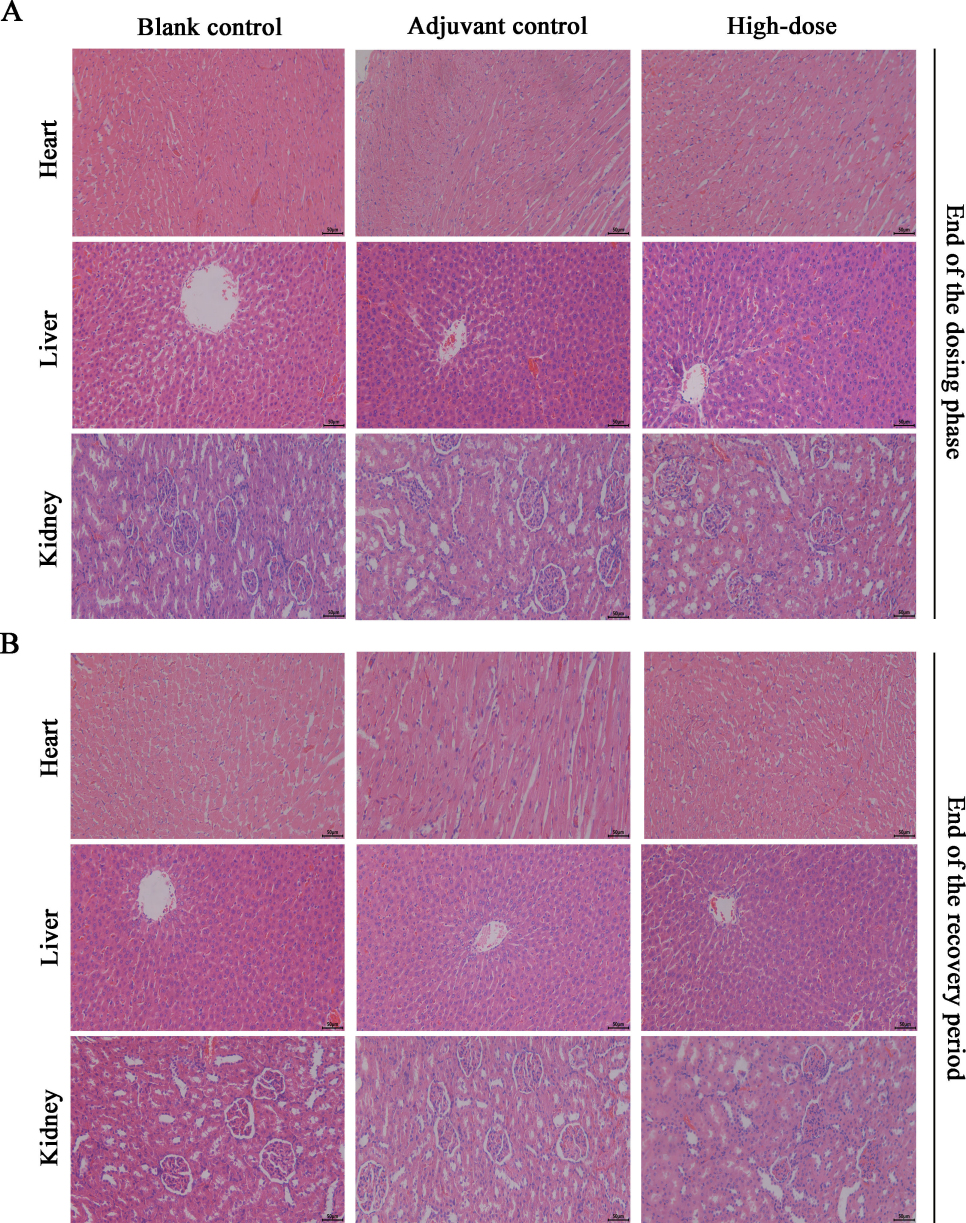
Histopathological results of major organs of ZF2001 after 3 doses of vaccination. **(A, B)** The H&E staining of the heart, liver, and kidney (200×, scale bar=100 μm), end of the dosing phase (n = 20), end of the recovery period (n = 10).

Necropsy and histopathology were performed on all the rats. At the examination of drug withdrawal, inflammatory reactions and foci of adjuvant deposition, lymph node hyperplasia and lymphatic sinus phagocytosis appeared at the site of administration in the rats of the adjuvant control group, the low-dose group and the high-dose group (**Figures 6A, C**), and these lesions were considered typical reactions to the localized administration of aluminum-containing adjuvants. At the end of the recovery period, changes in the site of administration of the three groups of rats and their lymph nodes still existed, but the extent of deposition at the site of administration and its degree were slightly lower. Histologic examination revealed obvious characteristics of chronic active inflammation, and all of the above changes were indicative of the recovery process (**Figures 6B, D**). In addition, the nondose-related microfocal lesions that appeared in some organs were considered to be background or spontaneous lesions that were unrelated to the administration of the vaccine. Furthermore, the results of pathological macroscopic and microscopic examination showed no histopathological changes related to the ZF2001 in any of the major tissues, such as the heart, liver, and kidney (representative images are shown in **Figure 7**).

## 4 Discussion

COVID-19 escalated into a global pandemic in 2020, remaining a major global public health threat and seriously affecting the lives of many people. Owing to the lack of effective therapies, safe and effective vaccination is still the optimum choice for controlling the epidemic and preventing COVID-19 infection. ZF2001, a protein subunit vaccine targeting the RBD of SARS-CoV-2, was shown to have good safety and efficacy in preventing and treating COVID-19 after three-dose vaccination. Therefore, a comprehensive assessment of the repeat-dose toxicity and safety of ZF2001 *in vivo* was necessary. In our laboratory, we conducted a series of preclinical safety evaluation studies in accordance with the Good Laboratory Practice of Nonclinical Studies (GLPs) (Turnheim, 1994; OECD, 1997). In this study, we evaluated the repeated-dose toxicity of ZF2001 in adult SD rats to determine the safety, tolerability and immunogenicity of the ZF2001 vaccine and to provide further support for its clinical application.

According to the data from clinical trials, the current recommended use of ZF2001 in the clinical setting is to administer three doses of 25 μg or 50 μg (Dai et al., 2022; Liao et al., 2022). In the present study, adult SD rats were injected with three doses of the ZF2001 vaccine at the same dosage used for humans (25 μg or 50 μg). The results revealed no significant abnormal reactions and no animal deaths occurred. In addition, there were no significant abnormalities in body weight, weight growth rate, food intake, urine index or ophthalmic examination of the rats after ZF2001 vaccination. These data suggest that the ZF2001 vaccine has no significant toxicological profile in terms of the general health status and clinical signs in rats.

Studies have shown that some COVID-19 vaccines may induce blood clotting, coagulation disorders and immune responses *in vivo (* O'Leary, 2021; Teijaro and Farber, 2021). In the present study, the hematological results revealed that PT was lower in the adjuvant, low-dose and high-dose groups than in the blank control group at the end of the treatment period. The levels of PT, APTT and Fbg in the adjuvant control and high-dose groups decreased at the end of the recovery period, whereas the %EOS in the high-dose group increased at the end of the treatment period and lasted until the end of the recovery period. The above changes in the reduction in coagulation-related indices (PT, APTT, Fbg) were considered related to the intramuscular injection of the aluminum-containing adjuvant, as the adjuvant control group also showed similar changes, with a smaller reduction in these indices. The %EOS of the high-dose group was greater at the end of the treatment and recovery periods than that of the adjuvant control group, but there was no significant difference from that of the blank control group, which might have been related to the inflammatory immune response of the body. Collectively, these results indicate that changes in the coagulation system and immune function should be closely monitored during the clinical application of the ZF2001 vaccine, which contains an aluminum adjuvant.

It has been reported that ZF2001 is a recombinant protein vaccine that induces high levels of RBD-binding and SARS-CoV-2 neutralizing antibodies, which effectively prevent and block the spread of COVID-19 (Xu et al., 2022). The results of phase 3 clinical trials in ZF2001 showed that it was 78.1% and 87.6% effective in preventing asymptomatic and severe-to-critical COVID-19, respectively, and that this protection could be sustained for at least 6 months (Dai et al., 2022). Our results revealed that at the end of the treatment period, the GLO level increased and the A/G ratio decreased in the high-dose group, which was consistent with the trend of increased IgG and C3 levels in the immunotoxicity test. All of the above abnormalities resolved at the end of the recovery period. The results of these immune-related indicators may be related to the enhanced immune response induced by ZF2001 vaccination. Notably, large-scale clinical trials have demonstrated that the ZF2001 vaccine is effective against COVID-19 and produces a broad-spectrum immune response against SARS-CoV-2 prototype strains and variants of concern, such as Delta, Omicron, BA.1 and BA.2 (Dai et al., 2022; Zhao et al., 2022). Similarly, the results of our immunogenicity test revealed that both male and female rats in the low-dose and high-dose groups produced high titers of anti-NCP-RBD IgG antibody *in vivo* after vaccine administration, indicating that the ZF2001 vaccine has good immunogenicity. Furthermore, based on current clinical reports, some COVID-19 vaccination could trigger an excessive immune response in some individuals, which may lead to myocarditis, autoimmune hepatitis or nephrotic syndrome (Unver et al., 2021; Boettler et al., 2022; Dong et al., 2022). However, our series studies conducted in rats and monkeys showed that the no pathologic damage to the heart, liver, kidneys, or other organs from the ZF2001 vaccine, suggesting that the ZF2001 may not elicit an excessive immune response (Yang et al., 2022). These results proved that ZF2001 vaccination leads to the production of antibodies that are highly immunogenic and persistent and that this antibody production is an amplification or extension of the pharmacological effects of ZF2001.

ZF2001, an aluminum hydroxide-adjuvanted SARS-CoV-2 recombinant RBD subunit vaccine, showed no clear dose-associated adverse effects that could be related to the intramuscular injection route or the aluminum hydroxide adjuvant (Meng et al., 2021). Aluminum hydroxide is the most commonly used vaccine adjuvant to enhance antigen-specific antibody responses and continue stimulating the immune system, and this adjuvant has been applied in licensed vaccines for hepatitis B and human papillomavirus infections (Egan et al., 2009; He et al., 2015). However, the occurrence of toxicity and side effects, including minor local reactions such as injection site pain and nodules, adjuvant arthritis, eosinophilia, sterile abscesses, eosinophilia, and myofascial pain, when aluminum hydroxide adjuvants are used at very high doses has raised widespread concern (Bansal et al., 2014; Tan et al., 2018). Indeed, the histopathological evaluation performed in this study suggested that the rats in the adjuvant, low-dose and high-dose groups presented significant inflammatory responses, adjuvant deposition lesions, lymph node hyperplasia and lymphatic sinus phagocytosis at the administration site during the end of the treatment period. Our data indicated that the above pathological changes in the adjuvant control group were similar to those in the high-dose group and slightly milder than those in the low-dose group and that these abnormal changes showed obvious recovery trends at the end of the recovery period. Moreover, in the adjuvant control and high-dose groups, the nodules could be palpated at the administration site after vaccination, and this effect lasted until the end of the recovery period. Importantly, no abnormal histopathological changes related to the ZF2001 vaccine were detected in other tissues or organs during the two periods. Considering the aluminum hydroxide adjuvant was used in both the ZF2001 vaccine injection and the adjuvant groups, we hypothesized that the above-described responses may have been related to the aluminum adjuvant and that the ZF2001 vaccination could be considered safe. Therefore, the results of this study suggest that the development and utilization of vaccines containing aluminum adjuvants should be thoroughly evaluated for *in vivo* safety and that it is necessary to develop a novel adjuvant-containing ZF2001 vaccine without side effects for immunocompromised populations.

In summary, ZF2001 was injected intramuscularly into rats at a dose of 25 or 50 μg NCP-RBD protein/dose for 3 times, which mainly caused the following changes: nodules at the administration site similar to those in the adjuvant group; effect on some blood biochemical indices (Na^+^↑, Cl^-^↑, PT↓, APTT↓, Fbg↓), inflammatory reactions at the administration site and adjuvant deposition; and lymph node proliferation and lymphatic sinus phagocytosis at the administration site (with a recovery trend observed during the recovery period). The above-listed abnormalities might have been related to the vaccination with an aluminum-containing adjuvant. This vaccination also had an influence on immune-related indices (%EOS↑, GLO↑, A/G↓, IgG↑, C3↑, spleen indices↑), which may have been related to the immune response after vaccination. Taken together, excluding the above adjuvant-related effects as well as immune responses, the results of this study indicate that the administration of ZF2001 by intramuscular injection yielded no significant toxic effects, there were no significant toxicity-targeted organs, and the no observed adverse effect level (NOAEL) was 50 μg NCP-RBD protein/rat.

## 5 Conclusion

The results of this study showed that ZF2001 vaccination causes no obvious adverse reactions in rats at the same dose as that used in humans. Moreover, this vaccine has good safety, tolerability and immunogenicity, as evidenced by the comprehensively evaluated repeated-dose toxicity of ZF2001. These findings provide a basis for the results of clinical trials.

## Data Availability

The original contributions presented in the study are included in the article/**Supplementary Material**. Further inquiries can be directed to the corresponding authors.
