# Supplementary material for: Repeated-dose toxicity and immunogenicity evaluation of a recombinant subunit COVID-19 vaccine (ZF2001) in rats
Source: Front Cell Infect Microbiol. 2025 Apr 22;15:1548787. doi: 10.3389/fcimb.2025.1548787 (PMC12053236; doi:10.3389/fcimb.2025.1548787)
Supplement: Supplementary file 1 [file Table1.docx]

**Supplementary data**

**Table S1. The list of abbreviations.**

| **Abbreviations** | | | |
| --- | --- | --- | --- |
| **A/G** | Albumin/Globulin | **LUC** | Large unstained cell |
| **ALB** | Albumin | **LYMPH** | Lymphocyte |
| **ALP** | Alkaline phosphatase | **MALB** | Microalbumin |
| **ALT** | Alanine aminotransferase | **MCH** | Mean corpusular hemoglobin |
| **APTT** | Activated partial thromboplastin time | **MCHC** | Mean corpusular hemoglobin concerntration |
| **AST** | Aspartate aminotransferase | **MCV** | Mean corpusular volume |
| **BASO** | Basophil | **MONO** | Monocyte |
| **BIL** | Bilirubin | **MPV** | Mean platelet volume |
| **BLD** | Blood | **Na** | Sodium |
| **BUN** | Blood urea nitrogen | **NEUT** | Neutrophil |
| **C3** | Complement 3 | **NIT** | Nitrite |
| **C4** | Complement 4 | **pH** | Potential of hydrogen |
| **Ca** | Calcium | **PLT** | Platelet |
| **CK** | Creatine kinase | **PRO** | Protein |
| **Cl** | Chlorine | **PT** | Prothrombin time |
| **Crea** | Crea | **RBC** | Red blood cell |
| **EOS** | Eosinophil | **RDW** | Red blood cell volume distribution width |
| **Fbg** | Fibrinogen | **RETIC** | Reticulocyte |
| **GLO** | Globulin | **SG** | Specific gravity |
| **GLU** | Glucose | **T.BIL** | Total Bilirubin |
| **HCT** | Hematocrit | **T.CHO** | Total Cholesterol |
| **HDW** | Hemoglobin distribution width | **T.P** | Total protein |
| **HGB** | Hemoglobin | **TG** | Total triglyceride |
| **IgG** | Immunoglobin G | **URO** | Urobilinogen |
| **K** | Potassium | **VC** | Vitamin C |
| **KET** | Ketone | **WBC** | White blood cell |

**Table S2. Test items of hematology, blood biochemical and urinalysis.**

| **Hematology** | | **Blood Biochemical** | **Urinalysis** |
| --- | --- | --- | --- |
| WBC（10e^3^/μL） | MCHC（g/L） | ALT（IU/L） | UBG |
| %NEUT（%） | RDW（%） | AST（IU/L） | BIL |
| %LYMPH（%） | HDW（g/L） | T.BIL（umol/L） | KET |
| %MONO（%） | MPV（fL） | ALP（IU/L） | BLD |
| %EOS（%） | PLT（10e^3^/μL） | CK（IU/L） | PRO |
| %BASO（%） | %RETIC（%） | T.P（g/L） | NIT |
| %LUC（%） | #RETIC（10e^3^/μL） | ALB（g/L） | WBC |
| #NEUT（10e^3^/μL） | PT（s） | GLO（g/L） | GLU |
| #LYMPH（10e^3^/μL） | Fbg（g/L） | A/G | MALB |
| #MONO（10e^3^/μL） | APTT（s） | GLU（mmol/L） | SG |
| #EOS（10e^3^/μL） |  | BUN（mmol/L） | pH |
| #BASO（10e^3^/μL） |  | Crea（umol/L） | VC |
| #LUC（10e^3^/μL） |  | T.CHO（mmol/L） |  |
| RBC（10e^6^/μL） |  | TG（mmol/L） |  |
| HGB（g/L） |  | K^+^（mmol/L） |  |
| HCT（%） |  | Na^+^（mmol/L） |  |
| MCV（fL） |  | Cl^-^（mmol/L） |  |
| MCH（pg） |  | Ca（mmol/L） |  |

**Table S3.** The body weight in SD rats treated with ZF001 vaccines in repeat-dose toxicity study.

| **Time** | **Male** | | | | **Female** | | | |
| --- | --- | --- | --- | --- | --- | --- | --- | --- |
|  | **Control** | **Adjuvant** | **Low-dose** | **High-Dose** | **Control** | **Adjuvant** | **Low-dose** | **High-dose** |
| Wf1 (*n*=15) | 303±12 | 292±16 | 298±14 | 291±19 | 208±10 | 203±12 | 198±10 | 207±11 |
| W1 (*n*=15) | 340±14 | 333±21 | 332±19 | 327±26 | 223±9 | 222±12 | 211±11**^△△^ | 219±10 |
| W2 (*n*=15) | 370±15 | 359±24 | 359±25 | 351±37 | 240±10 | 236±13 | 225 ± 14**^△^ | 235±12 |
| W3 (*n*=15) | 407±20 | 386±24 | 390±30 | 387±34 | 249±11 | 242±13 | 237±15 | 244±10 |
| W4 (*n*=15) | 419±18 | 406±30 | 407±32 | 406±37 | 262±14 | 257±17 | 250±15 | 259±12 |
| Wr1 (*n*=5) | 447±21 | 430±33 | 448±32 | 430±45 | 271±14 | 261±14 | 265±19 | 274±16 |
| Wr2 (*n*=5) | 470±18 | 449±35 | 468±32 | 453±44 | 284±19 | 273±8 | 271±19 | 283±17 |

**Note: Compared with the control group, **p*＜0.05, ***p*＜0.01. Compared with the adjuvant group,** ^△^ ***p*＜0.05,**^△△^ ***p*＜0.01.**

**Table S4.** The %change body weight in SD rats treated with ZF001 vaccines in repeat-dose toxicity study.

| **Time** | **Male** | | | | **Female** | | | |
| --- | --- | --- | --- | --- | --- | --- | --- | --- |
|  | **Control** | **Adjuvant** | **Low-dose** | **High-Dose** | **Control** | **Adjuvant** | **Low-dose** | **High-dose** |
| W1 (*n*=15) | 12.2±2.5 | 13.9±3.5 | 11.4±2.7 | 12.1±3.4 | 7.6±2.2 | 9.3±2.1 | 6.8±2.9^ΔΔ^ | 6.3±2.9^ΔΔ^ |
| W2 (*n*=15) | 22.2±3.6 | 22.8±4.7 | 20.4±4.9 | 20.3±7.6 | 15.8±5.4 | 15.9±3.6 | 13.7±2.5 | 13.8±3.0 |
| W3 (*n*=15) | 34.3±5.3 | 32.3±4.7 | 30.8±6.3 | 32.8±6.1 | 19.8±2.7 | 19.2±2.9 | 19.4±3.2 | 18.4±3.6 |
| W4 (*n*=15) | 38.4±4.9 | 39.1±6.4 | 36.7±6.7 | 39.1±6.5 | 25.9±3.8 | 26.5±3.5 | 26.0±3.0 | 25.4±3.8 |
| Wr1 (*n*=5) | 45.6±5.0 | 47.3±9.3 | 48.9±2.8 | 48.1±7.3 | 30.7±5.5 | 29.3±5.2 | 32.2±3.2 | 27.5±6.0 |
| Wr2 (*n*=5) | 53.2±5.4 | 53.8±10.3 | 55.8±3.0 | 56.1±7.5 | 36.7±6.7 | 35.4±3.8 | 35.4±3.8 | 32.1±6.5 |

**Note: Compared with the adjuvant group,** ^△^ ***p*＜0.05,** ^△△^ ***p*＜0.01.**

**Table S5.** The food consumption(g) in SD rats treated with ZF001 vaccines in repeat-dose toxicity study.

| **Time** | **Male** | | | | **Female** | | | |
| --- | --- | --- | --- | --- | --- | --- | --- | --- |
|  | **Control** | **Adjuvant** | **Low-dose** | **High-Dose** | **Control** | **Adjuvant** | **Low-dose** | **High-dose** |
| Wf1 (*cage*=3) | 24.1±0.5 | 22.1±1.8 | 24.9±1.0 | 23.5±2.2 | 16.3±0.8 | 15.8±0.5 | 15.9±1.5 | 15.3±0.4 |
| W1 (*cage*=3) | 27.7±0.5 | 26.9±1.4 | 25.5±1.8 | 25.4±0.9 | 18.2±1.5 | 18.3±0.9 | 17.8±1.6 | 17.3±0.3 |
| W2 (*cage*=3) | 28.9±0.8 | 26.8±2.9 | 26.1±2.3 | 27.1±1.5 | 19.5±0.8 | 19.3±1.1 | 17.2±0.7*^△^ | 18.9±0.8 |
| W3 (*cage*=3) | 27.5±0.5 | 25.7±1.1 | 25.6±0.7 | 26.0±1.4 | 18.3±1.1 | 16.9±0.1 | 17.9±1.3 | 17.3±1.8 |
| W4 (*cage*=3) | 25.4±1.0 | 23.3±1.6 | 24.4±0.9 | 23.6±0.9 | 17.4±0.5 | 16.1±0.6 | 17.1±0.9 | 16.7±1.8 |
| Wr1 (*cage*=2) | 24.2±3.1 | 23.8±0.4 | 27.4±0.1 | 25.0±1.4 | 23.4±12.8 | 21.6±2.7 | 20.9±2.0 | 20.8±1.1 |
| Wr2 (*cage*=2) | 26.0±1.4 | 25.6±0.1 | 25.6±1.5 | 26.4±1.3 | 20.3±8.8 | 18.8±0.4 | 16.4±1.5 | 19.3±2.4 |

**Note: Compared with the control group, **p*＜0.05, ***p*＜0.01. Compared with the adjuvant group,** ^△^ ***p*＜0.05,** ^△△^ ***p*＜0.01.**

**Table S6. Urinalysis in male rats before treatment period. (x̅±SD).**

| Group | URO umol/L | BIL umol/L | KET mmol/L | BLD Ery/ul | PRO g/L | NIT | WBC Leuko/ul | GLU mmol/L | MALB g/L | SG | pH | VC mmol/L |
| --- | --- | --- | --- | --- | --- | --- | --- | --- | --- | --- | --- | --- |
|  |  |  |  |  |  |  |  |  |  |  |  |  |
| Control | Normal：15/15 | Neg：15/15 | Neg：14/15  +-：1/15 | Neg：15/15 | Neg：15/15 | Neg：15/15 | Neg：15/15 | Neg：15/15 | Neg：1/15  0.15: 14/15 | 1.020: 7/15  1.025: 8/15 | 5.5: 9/15  6.0: 2/15  6.5: 3/15  7.0:1/15 | 0.0: 15/15 |
| Adjuvant | Normal：15/15 | Neg：15/15 | Neg：15/15  +-：0/15 | Neg：15/15 | Neg：14/15  Trace：1/15 | Neg：15/15 | Neg：15/15 | Neg：15/15 | Neg：9/15  0.15:5/15  >0.15: 1/15^*^ | 1.010: 2/15  1.015: 7/15  1.020: 1/15  1.025: 5/15 | 5.5: 2/15  6.0: 4/15  6.5: 3/15  7.0: 5/15  7.5: 1/15  ^*^ | 0.0: 15/15 |
| Low-dose | Normal：15/15 | Neg：15/15 | Neg：12/15  +-：3/15 | Neg：15/15 | Neg：15/15 | Neg：15/15 | Neg：10/15  +-：5/15  ^*△^ | Neg：15/15 | Neg：6/15  0.15: 9/15 | 1.015: 4/15  1.020:6/15  1.025: 5/15 | 5.5: 5/15  6.0: 5/15  6.5: 4/15  7.0: 1/15 | 0.0: 15/15 |
| High-dose | Normal：15/15 | Neg：15/15 | Neg：14/15  +-：1/15 | Neg：15/15 | Neg：14/15  Trace：1/15 | Neg：15/15 | Neg：13/15  +-：1/15  2+：1/15 | Neg：15/15 | Neg：3/15  0.15: 11/15  >0.15: 1/15 | 1.015: 3/15  1.020: 6/15  1.025: 6/15 | 5.5: 1/15  6.0: 5/15  6.5: 5/15  7.0: 2/15  7.5: 2/15  ^*^ | 0.0: 15/15 |

**Note: Compared with the control group, ^*^*p*＜0.05, ^**^*p*＜0.01. Compared with the adjuvant group,** ^△^ ***p*＜0.05,** ^△△^ ***p*＜0.01.**

**Table S7. Urinalysis in female rats before treatment period. (x̅±SD).**

| Group | URO umol/L | BIL umol/L | KET mmol/L | BLD Ery/ul | PRO g/L | NIT | WBC Leuko/ul | GLU mmol/L | MALB g/L | SG | pH | VC mmol/L |
| --- | --- | --- | --- | --- | --- | --- | --- | --- | --- | --- | --- | --- |
|  |  |  |  |  |  |  |  |  |  |  |  |  |
| Control | Normal：15/15 | Neg：15/15 | Neg：14/15  +-：1/15 | Neg：15/15 | Neg：14/15  Trace：1/15 | Neg：15/15 | Neg：14/15  2+：1/15 | Neg：15/15 | Neg：11/15  0.15: 3/15  >0.15: 1/15 | 1.015: 5/15  1.020: 4/15  1.025: 5/15  ≥1.030: 1/15 | 5.5: 3/15  6.0: 2/15  6.5: 6/15  7.0: 4/15 | 0.0: 10/15  0.6: 5/15 |
| Adjuvant | Normal：15/15 | Neg：15/15 | Neg：14/15  +-：1/15 | Neg：15/15 | Neg：14/15  Trace：1/15 | Neg：15/15 | Neg：14/15  1+: 1/15 | Neg：15/15 | Neg：7/15  0.15:7/15  >0.15: 1/15 | 1.015: 2/15  1.020: 5/15  1.025: 7/15  ≥1.030: 1/15 | 5.5: 1/15  6.0: 10/15  6.5: 3/15  7.0: 1/15 | 0.0: 9/15  0.6: 6/15 |
| Low-dose | Normal：15/15 | Neg：14/15  1+：1/15 | Neg：14/15  +-：1/15 | Neg：15/15 | Neg：14/15  Trace：1/15 | Neg：15/15 | Neg：12/15  1+: 1/15  +-：1/15  2+: 1/15 | Neg：15/15 | Neg：8/15  0.15: 5/15  >0.15: 2/15 | 1.015: 5/15  1.020: 4/15  1.025: 5/15  ≥1.030: 1/15 | 5.5: 1/15  6.0: 4/15  6.5: 8/15  7.0: 2/15 | 0.0: 13/15  0.6: 1/15  1.4: 1/15 |
| High-dose | Normal：15/15 | Neg：15/15 | Neg：15/15 | Neg：15/15 | Neg：15/15 | Neg：15/15 | Neg：15/15 | Neg：15/15 | Neg：9/15  0.15: 6/15 | 1.015: 5/15  1.020: 4/15  1.025: 5/15  ≥1.030: 1/15 | 6.0: 4/15  6.5: 4/15  7.0: 4/15  7.5: 3/15  ^△^ | 0.0: 13/15  0.6: 2/15 |

**Note: Compared with the control group, ^*^*p*＜0.05, ^**^*p*＜0.01. Compared with the adjuvant group,** ^△^ ***p*＜0.05,** ^△△^ ***p*＜0.01.**

**Table S8. Urinalysis in male rats after treatment period. (x̅±SD).**

| Group | URO umol/L | BIL umol/L | KET mmol/L | BLD Ery/ul | PRO g/L | NIT | WBC Leuko/ul | GLU mmol/L | MALB g/L | SG | pH | VC mmol/L |
| --- | --- | --- | --- | --- | --- | --- | --- | --- | --- | --- | --- | --- |
| Control | Normal：7/15  1+：8/15 | Neg：12/15  1+：3/15 | +-：1/15  1+：14/15 | Neg：15/15 | 1+：4/15  2+：11/15 | Neg：15/15 | +-：12/15  1+：3/15 | Neg：15/15 | >0.15：15/15 | 1.010：1/15  1.015：5/15  1.020：7/15  1.025：2/15 | 7.0：4/15  7.5：9/15  8.0：2/15 | 0.0：11/15  0.6： 3/15  2.8： 1/15 |
| Adjuvant | Normal：8/15  1+：7/15 | Neg：13/15  1+：2/15 | +-：3/15  1+：11/15  2+：1/15 | Neg：14/15  +-：1/15 | Trace：3/15  1+：6/15  2+：5/15  3+：1/15 | Neg：15/15 | Neg：1/15  +-：9/15  1+：5/15 | Neg：15/15 | >0.15：15/15 | ≤1.005：1/15  1.015：6/15  1.020：4/15  1.025：3/15  ≥1.030：1/15 | 6.5：2/15  7.0：3/15  7.5：5/15  8.0：4/15  8.5：1/15 | 0.0： 7/15  0.6： 2/15  1.4： 4/15  2.8： 2/15 |
| Low-dose | Normal：10/15  1+：5/15 | Neg：13/15  1+：2/15 | +-：6/15  1+：9/15 | Neg：15/15 | Neg：2/15  Trace：3/15  1+：6/15  2+：2/15  3+：2/15 | Neg：15/15 | Neg：2/15  +-：12/15  1+：1/15 | Neg：15/15 | 0.15：2/15  >0.15：13/15 | ≤1.005：1/15  1.010：3/15  1.015：4/15  1.020：5/15  1.025：2/15 | 6.0：1/15  6.5：1/15  7.0：4/15  7.5：8/15  8.5：1/15 | 0.0：12/15  1.4： 3/15 |
| High-dose | Normal：13/15  1+：2/15 | Neg：14/15  1+：1/15 | +-：7/15  1+：8/15 | Neg：13/15  3+：2/15 | Trace：1/15  1+：10/15  2+：4/15 | Neg：15/15 | Neg：1/15  +-：12/15  1+：2/15 | Neg：15/15 | >0.15：15/15 | 1.010：1/15  1.015：7/15  1.020：4/15  1.025：3/15 | 6.0：2/15  6.5：6/15  7.0：2/15  7.5：4/15  8.5：1/15 | 0.0：14/15  0.6： 1/15^△^ |

**Note: Compared with the control group, ^*^*p*＜0.05, ^**^*p*＜0.01. Compared with the adjuvant group,** ^△^ ***p*＜0.05,** ^△△^ ***p*＜0.01.**

**Table S9 Urinalysis in female rats after treatment period. (x̅±SD).**

| Group | URO umol/L | BIL umol/L | KET mmol/L | BLD Ery/ul | PRO g/L | NIT | WBC Leuko/ul | GLU mmol/L | MALB g/L | SG | pH | VC mmol/L |
| --- | --- | --- | --- | --- | --- | --- | --- | --- | --- | --- | --- | --- |
|  |  |  |  |  |  |  |  |  |  |  |  |  |
| Control | Normal：15/15 | Neg：15/15 | Neg：14/15  +-：1/15 | Neg：14/15  3+：1/15 | Neg：14/15  1+：1/15 | Neg：15/15 | Neg：8/15  1+: 7/15 | Neg：15/15 | Neg：12/15  0.15：2/15  >0.15：1/15 | ≤1.005：12/15  1.010：2/15  1.025：1/15 | 7.0: 2/15  7.5:11/15  8.0: 2/15 | 0.0: 15/15 |
| Adjuvant | Normal：15/15 | Neg：15/15 | Neg：15/15 | Neg：13/15  +-：1/15  1+：1/15 | Neg：15/15 | Neg：15/15 | Neg：6/15  +-：1/15  1+: 8/15 | Neg：15/15 | Neg：12/15  0.15：3/15 | ≤1.005：12/15  1.010：2/15  1.015：1/15 | 6.5: 3/15  7.0:1/15  7.5:9/15  8.0:1/15  8.5: 1/15 | 0.0: 15/15 |
| Low-dose | Normal：15/15 | Neg：14/15  1+：1/15 | Neg：14/15  +-：1/15 | Neg：15/15 | Neg：14/15  1+：1/15 | Neg：15/15 | Neg：8/15  +-：1/15  1+: 6/15 | Neg：15/15 | Neg：8/15  0.15：6/15  >0.15：1/15 | ≤1.005：10/15  1.010：2/15  1.015：2/15  1.025：1/15 | 6.5: 3/15  7.0:2/15  7.5:4/15  8.0:6/15 | 0.0: 15/15 |
| High-dose | Normal：15/15 | Neg：15/15 | Neg：15/15 | Neg：14/15  +-：1/15 | Neg：15/15 | Neg：15/15 | Neg：11/15  +-：1/15  1+: 3/15 | Neg：15/15 | Neg：14/15  0.15：1/15 | ≤1.005：13/15  1.010：2/15 | 7.0:2/15  7.5:10/15  8.0:3/15 | 0.0: 15/15 |

**Note: Compared with the control group, ^*^*p*＜0.05, ^**^*p*＜0.01. Compared with the adjuvant group,** ^△^ ***p*＜0.05,** ^△△^ ***p*＜0.01.**

**Table S10. Urinalysis in male rats after recovery period. (x̅±SD).**

| Group | URO umol/L | BIL umol/L | KET mmol/L | BLD Ery/ul | PRO g/L | NIT | WBC Leuko/ul | GLU mmol/L | MALB g/L | SG | pH | VC mmol/L |
| --- | --- | --- | --- | --- | --- | --- | --- | --- | --- | --- | --- | --- |
| Control | Normal：5/5 | Neg：5/5 | Neg：5/5 | Neg：5/5 | Neg：5/5 | Neg：5/5 | Neg：5/5 | Neg：5/5 | Neg：5/5 | <=1.005：4/5  1.010：1/5 | 6.0：1/5  6.5：1/5  7.5：3/5 | 0.0：5/5 |
| Adjuvant | Normal：5/5 | Neg：5/5 | Neg：5/5 | Neg：5/5 | Neg：5/5 | Neg：5/5 | Neg：5/5 | Neg：5/5 | Neg：5/5 | <=1.005：4/5  1.010：1/5 | 6.5：1/5  7.0：3/5  7.5：1/5 | 0.0：5/5 |
| Low-dose | Normal：5/5 | Neg：5/5 | Neg：4/5  +-：1/5 | Neg：5/5 | Neg：5/5 | Neg：5/5 | Neg：5/5 | Neg：5/5 | Neg：5/5 | <=1.005：4/5  1.010：1/5 | 6.0：2/5  7.5：2/5  8.0：1/5 | 0.0：5/5 |
| High-dose | Normal：5/5 | Neg：5/5 | Neg：5/5 | Neg：5/5 | Neg：5/5 | Neg：5/5 | Neg：4/5  +-：1/5 | Neg：5/5 | Neg：5/5 | <=1.005：4/5  1.010：1/5 | 7.5：5/5 | 0.0：5/5 |

**Note: Compared with the control group, ^*^*p*＜0.05, ^**^*p*＜0.01. Compared with the adjuvant group,** ^△^ ***p*＜0.05,** ^△△^ ***p*＜0.01.**

**Table S11. Urinalysis in female rats after recovery period. (x̅±SD).**

| Group | URO umol/L | BIL umol/L | KET mmol/L | BLD Ery/ul | PRO g/L | NIT | WBC Leuko/ul | GLU mmol/L | MALB g/L | SG | pH | VC mmol/L |
| --- | --- | --- | --- | --- | --- | --- | --- | --- | --- | --- | --- | --- |
| Control | Normal：5/5 | Neg：5/5 | Neg：1/5  +-：4/5 | Neg：5/5 | Neg：5/5 | Neg：5/5 | Neg：1/5  +-：3/5  1+：1/5 | Neg：5/5 | Neg：5/5 | <=1.005：4/5  1.015：1/5 | 6.5：1/5  7.0：1/5  7.5：3/5 | 0.0：5/5 |
| Adjuvant | Normal：5/5 | Neg：5/5 | Neg：2/5  +-：3/5 | Neg：4/5  1+：1/5 | Neg：5/5 | Neg：5/5 | Neg：2/5  +-：3/5 | Neg：5/5 | Neg：5/5 | <=1.005：5/5 | 7.5：4/5  8.0：1/5 | 0.0：5/5 |
| Low-dose | Normal：5/5 | Neg：5/5 | Neg：2/5  +-：3/5 | Neg：5/5 | Neg：4/5  Trace：1/5 | Neg：5/5 | Neg：1/5  +-：4/5 | Neg：5/5 | Neg：4/5  >0.15：1/5 | <=1.005：5/5 | 6.5：1/5  7.0：1/5  7.5：3/5 | 0.0：5/5 |
| High-dose | Normal：5/5 | Neg：5/5 | Neg：1/5  +-：4/5 | Neg：5/5 | Neg：4/5  Trace：1/5 | Neg：5/5 | +-：5/5 | Neg：5/5 | Neg：4/5  >0.15：1/5 | <=1.005：1/5  1.010：4/5  ^△^ | 7.5：4/5  8.0：1/5 | 0.0：5/5 |

**Note: Compared with the control group, ^*^*p*＜0.05, ^**^*p*＜0.01. Compared with the adjuvant group,** ^△^ ***p*＜0.05,** ^△△^ ***p*＜0.01.**

**Table S12. Organ weight and organ coefficients in male rat after treatment period. (x̅±SD).**

| **Test item** | | **Group** | | | | | | | | | | | |
| --- | --- | --- | --- | --- | --- | --- | --- | --- | --- | --- | --- | --- | --- |
|  |  | **Blank control** | | | **Adjuvant control** | | | **Low-dose** | | | **High-dose** | | |
| **Animals, *n*** | | **10** | | | **10** | | | **10** | | | **10** | | |
| **Brain** | Weight (g) | 2.125 | ± | 0.061 | 2.112 | ± | 0.094 | 2.145 | ± | 0.084 | 2.137 | ± | 0.073 |
|  | Organ coefficient (%) | 0.523 | ± | 0.029 | 0.542 | ± | 0.043 | 0.554 | ± | 0.052 | 0.552 | ± | 0.054 |
| **Heart** | Weight (g) | 1.370 | ± | 0.112 | 1.289 | ± | 0.095 | 1.301 | ± | 0.124 | 1.329 | ± | 0.099 |
|  | Organ coefficient (%) | 0.336 | ± | 0.019 | 0.330 | ± | 0.022 | 0.334 | ± | 0.018 | 0.343 | ± | 0.036 |
| **Liver** | Weight (g) | 12.600 | ± | 1.522 | 11.466 | ± | 0.920 | 11.896 | ± | 1.793 | 12.124 | ± | 1.594 |
|  | Organ coefficient (%) | 3.089 | ± | 0.266 | 2.931 | ± | 0.128 | 3.043 | ± | 0.284 | 3.104 | ± | 0.270 |
| **Spleen** | Weight (g) | 0.845 | ± | 0.129 | 0.881 | ± | 0.074 | 0.884 | ± | 0.087 | 0.921 | ± | 0.094 |
|  | Organ coefficient (%) | 0.207 | ± | 0.025 | 0.225 | ± | 0.017 | 0.228 | ± | 0.021^*^ | 0.237 | ± | 0.025^**^ |
| **Kidney** | Weight (g) | 3.066 | ± | 0.254 | 3.033 | ± | 0.252 | 2.963 | ± | 0.308 | 3.034 | ± | 0.303 |
|  | Organ coefficient (%) | 0.754 | ± | 0.064 | 0.776 | ± | 0.049 | 0.760 | ± | 0.043 | 0.779 | ± | 0.056 |
| **Thymus** | Weight (g) | 0.460 | ± | 0.169 | 0.425 | ± | 0.072 | 0.441 | ± | 0.090 | 0.461 | ± | 0.136 |
|  | Organ coefficient (%) | 0.113 | ± | 0.039 | 0.109 | ± | 0.018 | 0.114 | ± | 0.024 | 0.118 | ± | 0.029 |
| **Adrenal gland** | Weight (g) | 0.066 | ± | 0.009 | 0.060 | ± | 0.006 | 0.061 | ± | 0.008 | 0.062 | ± | 0.010 |
|  | Organ coefficient (%) | 0.016 | ± | 0.002 | 0.015 | ± | 0.001 | 0.016 | ± | 0.002 | 0.016 | ± | 0.003 |
| **Testicle** | Weight (g) | 3.741 | ± | 0.271 | 3.750 | ± | 0.413 | 3.684 | ± | 0.223 | 3.739 | ± | 0.213 |
|  | Organ coefficient (%) | 0.921 | ± | 0.074 | 0.959 | ± | 0.090 | 0.951 | ± | 0.095 | 0.965 | ± | 0.104 |
| **Epididymis** | Weight (g) | 1.177 | ± | 0.106 | 1.192 | ± | 0.122 | 1.202 | ± | 0.066 | 1.203 | ± | 0.068 |
|  | Organ coefficient (%) | 0.290 | ± | 0.030 | 0.305 | ± | 0.026 | 0.311 | ± | 0.033 | 0.311 | ± | 0.035 |

**Note: Compared with the control group, ^*^*p*＜0.05, ^**^*p*＜0.01. Compared with the adjuvant group,** ^△^ ***p*＜0.05,** ^△△^ ***p*＜0.01.**

**Table S13. Organ weight and organ coefficients in female rats after treatment period. (x̅±SD).**

| **Test item** | | **Group** | | | | | | | | | | | |
| --- | --- | --- | --- | --- | --- | --- | --- | --- | --- | --- | --- | --- | --- |
|  |  | **Blank control** | | | **Adjuvant control** | | | **Low-dose** | | | **High-dose** | | |
| **Animals, *n*** | | **10** | | | **10** | | | **10** | | | **10** | | |
| **Brain** | Weight (g) | 1.998 | ± | 0.049 | 2.045 | ± | 0.078 | 2.026 | ± | 0.068 | 1.987 | ± | 0.094 |
|  | Organ coefficient (%) | 0.816 | ± | 0.049 | 0.829 | ± | 0.048 | 0.876 | ± | 0.063 | 0.822 | ± | 0.052 |
| **Heart** | Weight (g) | 0.864 | ± | 0.083 | 0.883 | ± | 0.066 | 0.863 | ± | 0.123 | 0.895 | ± | 0.088 |
|  | Organ coefficient (%) | 0.352 | ± | 0.024 | 0.358 | ± | 0.025 | 0.372 | ± | 0.046 | 0.370 | ± | 0.030 |
| **Liver** | Weight (g) | 7.732 | ± | 0.585 | 7.790 | ± | 0.640 | 7.433 | ± | 0.373 | 7.729 | ± | 0.522 |
|  | Organ coefficient (%) | 3.148 | ± | 0.148 | 3.148 | ± | 0.160 | 3.210 | ± | 0.181 | 3.194 | ± | 0.219 |
| **Spleen** | Weight (g) | 0.617 | ± | 0.068 | 0.647 | ± | 0.049 | 0.625 | ± | 0.060 | 0.653 | ± | 0.095 |
|  | Organ coefficient (%) | 0.251 | ± | 0.025 | 0.262 | ± | 0.023 | 0.270 | ± | 0.032 | 0.269 | ± | 0.035 |
| **Kidney** | Weight (g) | 1.896 | ± | 0.084 | 1.936 | ± | 0.187 | 1.884 | ± | 0.120 | 1.964 | ± | 0.087 |
|  | Organ coefficient (%) | 0.773 | ± | 0.033 | 0.782 | ± | 0.042 | 0.813 | ± | 0.045 | 0.812 | ± | 0.048 |
| **Thymus** | Weight (g) | 0.387 | ± | 0.092 | 0.324 | ± | 0.068* | 0.303 | ± | 0.038^**^ | 0.341 | ± | 0.051 |
|  | Organ coefficient (%) | 0.158 | ± | 0.037 | 0.132 | ± | 0.030 | 0.131 | ± | 0.014 | 0.141 | ± | 0.021 |
| **Adrenal gland** | Weight (g) | 0.069 | ± | 0.006 | 0.070 | ± | 0.009 | 0.069 | ± | 0.010 | 0.069 | ± | 0.009 |
|  | Organ coefficient (%) | 0.028 | ± | 0.002 | 0.028 | ± | 0.003 | 0.030 | ± | 0.004 | 0.028 | ± | 0.004 |
| **Uterus** | Weight (g) | 0.696 | ± | 0.256 | 0.693 | ± | 0.161 | 0.630 | ± | 0.144 | 0.728 | ± | 0.275 |
|  | Organ coefficient (%) | 0.282 | ± | 0.095 | 0.281 | ± | 0.068 | 0.270 | ± | 0.050 | 0.303 | ± | 0.123 |
| **Ovary** | Weight (g) | 0.118 | ± | 0.015 | 0.109 | ± | 0.021 | 0.111 | ± | 0.028 | 0.116 | ± | 0.030 |
|  | Organ coefficient (%) | 0.048 | ± | 0.005 | 0.044 | ± | 0.009 | 0.048 | ± | 0.012 | 0.048 | ± | 0.012 |

**Note: Compared with the control group, ^*^*p*＜0.05, ^**^*p*＜0.01. Compared with the adjuvant group,** ^△^ ***p*＜0.05,** ^△△^ ***p*＜0.01.**

**Table S14. Organ weight and organ coefficients in male rats after recovery period. (x̅±SD).**

| **Test item** | | **Group** | | | | | | | | | | | |
| --- | --- | --- | --- | --- | --- | --- | --- | --- | --- | --- | --- | --- | --- |
|  |  | **Blank control** | | | **Adjuvant control** | | | **Low-dose** | | | **High-dose** | | |
| **Animals, *n*** | | **5** | | | **5** | | | **5** | | | **5** | | |
| **Brain** | Weight (g) | 2.153 | ± | 0.049 | 2.213 | ± | 0.081 | 2.200 | ± | 0.092 | 2.295 | ± | 0.038** |
|  | Organ coefficient (%) | 0.488 | ± | 0.023 | 0.520 | ± | 0.036 | 0.497 | ± | 0.027 | 0.545 | ± | 0.060 |
| **Heart** | Weight (g) | 1.402 | ± | 0.077 | 1.447 | ± | 0.133 | 1.413 | ± | 0.094 | 1.387 | ± | 0.115 |
|  | Organ coefficient (%) | 0.318 | ± | 0.023 | 0.339 | ± | 0.029 | 0.319 | ± | 0.013 | 0.329 | ± | 0.044 |
| **Liver** | Weight (g) | 12.095 | ± | 0.857 | 12.388 | ± | 1.228 | 12.417 | ± | 1.408 | 11.831 | ± | 1.194 |
|  | Organ coefficient (%) | 2.734 | ± | 0.126 | 2.895 | ± | 0.102 | 2.794 | ± | 0.204 | 2.787 | ± | 0.152 |
| **Spleen** | Weight (g) | 0.938 | ± | 0.074 | 0.890 | ± | 0.070 | 0.930 | ± | 0.063 | 0.939 | ± | 0.121 |
|  | Organ coefficient (%) | 0.212 | ± | 0.014 | 0.208 | ± | 0.025 | 0.210 | ± | 0.020 | 0.221 | ± | 0.018 |
| **Kidney** | Weight (g) | 3.280 | ± | 0.338 | 3.324 | ± | 0.613 | 3.393 | ± | 0.464 | 3.231 | ± | 0.375 |
|  | Organ coefficient (%) | 0.741 | ± | 0.048 | 0.773 | ± | 0.089 | 0.763 | ± | 0.071 | 0.760 | ± | 0.039 |
| **Thymus** | Weight (g) | 0.464 | ± | 0.039 | 0.368 | ± | 0.100 | 0.415 | ± | 0.103 | 0.371 | ± | 0.050 |
|  | Organ coefficient (%) | 0.105 | ± | 0.006 | 0.086 | ± | 0.021 | 0.094 | ± | 0.025 | 0.089 | ± | 0.020 |
| **Adrenal gland** | Weight (g) | 0.060 | ± | 0.011 | 0.072 | ± | 0.011 | 0.067 | ± | 0.006 | 0.064 | ± | 0.005 |
|  | Organ coefficient (%) | 0.014 | ± | 0.003 | 0.017 | ± | 0.001 | 0.015 | ± | 0.002 | 0.015 | ± | 0.002 |
| **Testicle** | Weight (g) | 3.833 | ± | 0.269 | 3.701 | ± | 0.557 | 3.813 | ± | 0.393 | 3.768 | ± | 0.300 |
|  | Organ coefficient (%) | 0.867 | ± | 0.058 | 0.865 | ± | 0.100 | 0.862 | ± | 0.096 | 0.895 | ± | 0.129 |
| **Epididymis** | Weight (g) | 1.296 | ± | 0.087 | 1.368 | ± | 0.126 | 1.394 | ± | 0.084 | 1.340 | ± | 0.053 |
|  | Organ coefficient (%) | 0.293 | ± | 0.017 | 0.320 | ± | 0.022 | 0.314 | ± | 0.008 | 0.318 | ± | 0.034 |

**Note: Compared with the control group, ^*^*p*＜0.05, ^**^*p*＜0.01. Compared with the adjuvant group,** ^△^ ***p*＜0.05,** ^△△^ ***p*＜0.01.**

**Table S15. Organ weight and organ coefficients in female rats after recovery period. (x̅±SD).**

| **Test item** | | **Group** | | | | | | | | | | | |
| --- | --- | --- | --- | --- | --- | --- | --- | --- | --- | --- | --- | --- | --- |
|  |  | **Control** | | | **Adjuvant** | | | **Low-dose** | | | **High-dose** | | |
| **Animals, *n*** | | **5** | | | **5** | | | **5** | | | **5** | | |
| **Brain** | Weight (g) | 2.090 | ± | 0.024 | 2.031 | ± | 0.065 | 2.017 | ± | 0.080 | 2.065 | ± | 0.108 |
|  | Organ coefficient (%) | 0.798 | ± | 0.050 | 0.806 | ± | 0.023 | 0.804 | ± | 0.063 | 0.777 | ± | 0.052 |
| **Heart** | Weight (g) | 0.940 | ± | 0.122 | 0.911 | ± | 0.037 | 0.937 | ± | 0.091 | 0.913 | ± | 0.054 |
|  | Organ coefficient (%) | 0.357 | ± | 0.032 | 0.362 | ± | 0.026 | 0.372 | ± | 0.020 | 0.344 | ± | 0.030 |
| **Liver** | Weight (g) | 7.580 | ± | 0.248 | 7.702 | ± | 0.465 | 7.134 | ± | 0.754 | 7.297 | ± | 0.363 |
|  | Organ coefficient (%) | 2.889 | ± | 0.114 | 3.059 | ± | 0.239 | 2.827 | ± | 0.089^△^ | 2.745 | ± | 0.132^△△^ |
| **Spleen** | Weight (g) | 0.665 | ± | 0.115 | 0.622 | ± | 0.049 | 0.653 | ± | 0.098 | 0.654 | ± | 0.103 |
|  | Organ coefficient (%) | 0.253 | ± | 0.036 | 0.246 | ± | 0.012 | 0.259 | ± | 0.032 | 0.247 | ± | 0.042 |
| **Kidney** | Weight (g) | 2.012 | ± | 0.107 | 2.000 | ± | 0.147 | 1.940 | ± | 0.203 | 2.058 | ± | 0.143 |
|  | Organ coefficient (%) | 0.769 | ± | 0.077 | 0.793 | ± | 0.047 | 0.770 | ± | 0.057 | 0.773 | ± | 0.037 |
| **Thymus** | Weight (g) | 0.331 | ± | 0.102 | 0.287 | ± | 0.037 | 0.258 | ± | 0.022 | 0.305 | ± | 0.100 |
|  | Organ coefficient (%) | 0.125 | ± | 0.034 | 0.113 | ± | 0.011 | 0.103 | ± | 0.011 | 0.115 | ± | 0.039 |
| **Adrenal**  **gland** | Weight (g) | 0.077 | ± | 0.004 | 0.072 | ± | 0.010 | 0.074 | ± | 0.007 | 0.072 | ± | 0.008 |
|  | Organ coefficient (%) | 0.029 | ± | 0.002 | 0.029 | ± | 0.004 | 0.029 | ± | 0.003 | 0.027 | ± | 0.003 |
| **Uterus** | Weight (g) | 0.871 | ± | 0.194 | 0.680 | ± | 0.345 | 0.556 | ± | 0.065 | 0.771 | ± | 0.291 |
|  | Organ coefficient (%) | 0.334 | ± | 0.089 | 0.271 | ± | 0.141 | 0.221 | ± | 0.026 | 0.288 | ± | 0.104 |
| **Ovary** | Weight (g) | 0.120 | ± | 0.018 | 0.112 | ± | 0.018 | 0.105 | ± | 0.013 | 0.100 | ± | 0.013 |
|  | Organ coefficient (%) | 0.046 | ± | 0.008 | 0.045 | ± | 0.008 | 0.042 | ± | 0.005 | 0.038 | ± | 0.006 |

**Note: Compared with the control group, ^*^*p*＜0.05, ^**^*p*＜0.01. Compared with the adjuvant group,** ^△^ ***p*＜0.05,** ^△△^ ***p*＜0.01.**
